# Lawson Wilkins and my life: part 3

**DOI:** 10.1186/1687-9856-2014-S1-S4

**Published:** 2014-05-28

**Authors:** Claude J Migeon

**Affiliations:** 1 502 Somerset Rd, Baltimore, MD 21210, USA

## The legacy of Lawson Wilkins: a new medical specialty, pediatric endocrinology

At this point, it is important to outline the contributions of Dr. Wilkins to the medical field. He is often referred to as the “Father of Pediatric Endocrinology.” Indeed, he came to the field of Pediatrics at a time when it was subdivided into specialties. He and his textbook “The Diagnosis and Treatment of Endocrine Disorders in Childhood and Adolescence” (1^st^ Edition, 1950) were the basis of this new field.

How he came to this is well presented by one of his early fellows, Judson J. Van Wyk who gave a conference on the subject in October 28, 2003, and is reported here as follows:

### Tale of Lawson Wilkins By Judson Van Wyk October 28, 2003

Many were surprised to learn that Dr. Wilkins was a general pediatrician in private practice until he was over 50. Wilkins’s father was a general practitioner in Baltimore whose hero was William Osler. Lawson sometimes drove the horse and buggy for his father on his rounds and later cited his Dad as one of the 3 most important influences on his career, the others being Fuller Albright and Edwards Park.

Lawson attended Johns Hopkins Medical School, but received his MD in France, where he had spent his 4^th^ year as an orderly on the battlefields of World War I. On his return he took a medical internship at Yale and a pediatric residency in Pediatrics at the Harriett Lane Home under John Howland.

Wilkins’s solo practice as a private pediatrician in Baltimore gave him the freedom to spend part of each week in one of the specialty clinics: such as the syphilis clinic or the epilepsy clinic. His first paper in 1923 was on the potassium content of human serum, carried out by a laborious gravimetric assay method.

(He was also interested in calcium metabolism and rickets. He wrote a few early papers in collaboration with Drs. Orr WJ, Holt LE Jr, Boone FH and Kramer, B.)

In 1935, Dr. Edwards Park, chief of Pediatrics at the Harriett Lane Home had the wisdom to foresee that the new discipline of endocrinology might be of value in understanding the growth and development of children; He showed even greater wisdom by asking Wilkins to organize a pediatric endocrine clinic. Wilkins resisted: “Do you want to make a charlatan out of me?”

Nevertheless, Wilkins accepted the challenge and spent every evening into the small hours reading the fat tomes written by so-called “experts” in the field. Wilkins became increasingly frustrated by the absence of any science and the long convoluted descriptions of endless “glandular syndromes.” Lawson was never one to suffer fools gladly, and his most important act one night while reading in bed was to fling the volume against the wall while cursing the stuffed shirts who could write such garbage.

He decided to begin over from scratch and learn everything he could by carefully documenting every clinical feature of his patients, and exploiting every opportunity to learn about mechanisms. Wilkins had little knowledge of statistics, but had a penetrating eye for what each of his patients could teach him. He had long ago adopting the practice of plotting all of his patients’ findings graphically as a function of time in the hope that such graphs might provide correlations and insights that might otherwise be missed.

Lacking centile growth charts as we now know them, Lawson adopted the 1932 charts of Engelbach which gave average values for age of height, weight, span, segmental proportions, and circumferences of the head, chest, and abdomen. He adopted these charts because they permitted correlations of body proportions with growth parameters.

He also laboriously traced out the appearance of epiphyses in the wrist, elbow, shoulder, knee, and ankle and developed his own scheme for determining bone age long before Greulich’s Atlas based on wrist x-rays.

### Hypothyroidism

He started by studying hypothyroidism, plotting the effect of thyroid substitution treatment and its withdrawal on parameters of growth and development. His 1938 paper [1] was his first contribution of the effect of an endocrine deficiency on growth. In it, he charted chronologic age versus the different measures of developmental age. He concluded from his many such graphs that bone maturation was the most sensitive indicator of adequate substitution therapy.

Wilkins recognized, however, that to understand the physiological effects of thyroid and other hormones, he had to turn to the laboratory, a task for which he was ill-prepared. But he was a good judge of talent and had the good fortune to meet Walter Fleischmann, a refugee Viennese physiologist. When the Journal of Clinical Endocrinology made its debut in 1941, the first 2 papers in each of the first 2 issues were by Wilkins and Fleischmann reporting their studies in hypothyroidism. [2-5]

Since there were no methods available to measure thyroid hormone levels directly, he measured the effects of thyroid on lowering the elevated cholesterol in hypothyroid children and raising the depressed levels of creatine in the urine. He found these measures far more reliable than measurements of basal metabolic rate.

It should come as no surprise, therefore, to learn that Wilkins had one of the first binary counters in Baltimore to study thyroid problems with radioactive iodine uptake studies, and one of the first to order the new protein bound iodine measurements on his patients.

In 1944, Wilkins went through a period of despondency following the tragic death of his only son. He was rescued by Dr. Park, who induced him to become a full time member of the Johns Hopkins Department of Pediatrics.

Wilkins brought with him his vast collection of case histories and photographs. He presented this extensive collection in a gigantic poster at the 1^st^ International Congress of Pediatrics in Zurich in 1950.

He then collated this material into the first Textbook of Pediatric Endocrinology. This launched, his fame spread rapidly, and students from the US and many foreign countries flocked to study under him. His textbook was considered the “Bible” of the field at the time.

### Congenital adrenal hyperplasia

Wilkins was particularly challenged by difficulties encountered by patients with congenital adrenal hyperplasia. He knew that the secretions of adrenal androgens were controlled by ACTH and he made several unsuccessful attempts to suppress ACTH secretion by administering biologically weak androgens. When cortisone became available for experimental use, Wilkins immediately saw it as a more likely participant in the ACTH feedback mechanism, and in short order was able to show that the secretion of adrenal androgens in girls with CAH could be inhibited by the administration of a glucocorticoid hormone.[6] Bartter and Albright in Boston made the same observation independently. Crigler and other postdoctoral fellows followed these initial reports with a series of classic studies that provided the essential data for long term adrenal suppression of these patients.[7]

Wilkins thought that the identification of steroid precursors in the urine of patients with CAH might reveal to us the biosynthetic pathways of human adrenal steroid biogenesis. Most steroid biochemists, however, were preoccupied with perfusing bovine adrenals with radio-labeled precursors and could not be bothered by clinical problems. Eventually he enlisted Dr. Seymour Lieberman in this endeavor and Lieberman accepted Wilkins’s prospective research associate, Dr. Alfred Bongiovanni, into his laboratory to learn advanced steroid methodology. As Wilkins had predicted, the studies of Bongiovanni led to the definition of the crucial enzymatic lesions in the different forms of adrenal hyperplasia. [8]

Al pioneered the pregnantriol method for monitoring adrenal suppression, and contributed greatly to our knowledge of the enzymatic defects in the several forms of CAH. Al and Walter Eberlein described and identified the defect in 11β-hydroxylase deficiency.

### Syndrome of gonadal dysgenesis and sex differentiation

Another early interest of Wilkins was sexual development. He was particularly intrigued by the syndrome of short stature, sexual infantilism, and multiple congenital anomalies in girls. This syndrome was not uncommon, and Henry Turner’s description had previously been described by a number of other authors. In 1942, 2 groups, Varney, Kenyon, and Koch in Chicago and Albright and his coworkers in Boston, reported that these girls had elevated urinary gonadotropins, thus demonstrating that their sexual infantilism was due to primary gonadal failure rather than hypopituitarism as had been suggested by Henry Turner in 1938. To learn more about their gonadal failure Wilkins persuaded Richard Te Linde, the chief of gynecology to explore the pelvis in 5 such patients.

He found that the gonads in all patients consisted only of fibrous streaks composed exclusively of ovarian stromal cells.

There were no germ cells or ovarian follicles, although there were occasional mesonephric remnants.

In his classic 1944 paper, Wilkins reported his findings and pointed out that studies of patients with streak gonads might provide powerful evidence supporting or refuting the various theories of sex differentiation. He critically evaluated each of the theories that had been advanced, including that of Weisner who believed that in the absence of fetal gonadal secretions all embryos would be feminine, although androgen could influence male differentiation.

### 1949 meeting with Alfred Jost

The classic studies of Jost on the fetal castration in rabbits are very well known now. They demonstrated that the fetal testis is necessary for differentiation as a male, but in the absence of fetal gonads both the internal and external genitalia developed as a normal female. In 1949, Jost presented his findings on sex differentiation to an International Congress of Gynecology in Mexico City. On the way home, he arranged to visit the Carnegie Institute of Embryology in Baltimore which housed Streeter and Corner, the leading human embryologists of the day. After hearing Jost’s story, the embryologists insisted that he meet Dr. Wilkins who had a healthy interest in the hormonal control of sex differentiation.

Twenty-five years later, Jost recalled the encounter:

“He was 55 and I was 33 and not even a doctor of medicine. He calmly and patiently followed my description of the rabbit experiments and asked many penetrating questions. He then showed me his portfolio of clinical cases of sexual ambiguity asking me to help him interpret the pathophysiology. The discussions went on for many hours into the late afternoon and Dr. Wilkins finally concluded, “I am convinced that if what you say is true, half of my beautiful girls with ovarian agenesis are really boys!”

Unfortunately there was no way to determine genetic sex at that time. Nevertheless, Wilkins felt sufficiently secure with this interpretation that he cited this hypothesis as a footnote in his 1950 textbook.

Four or five years after Lawson’s encounter with Alfred Jost, when Mel Grumbach and I were fellows of Wilkins, we reported in journal club that a Canadian pathologist, named Murray Barr had discovered a cytological marker in nuclei of female cells but not in male cells. Lawson immediately phone Murray Barr and arranged for him to analyze skin biopsies from a group of our patients with what we then called ovarian agenesis.

In 1954, we reported that 6 of 8 patients lacked the chromatin dot and presumably were “genetic XY males.”[9] Grumbach followed up on this and became a world authority on sex differentiation. [10]

In the 1960s, Malcolm Ferguson-Smith and Barbara Migeon did the karyotypes of the patients showing that most of them had only one X-chromosome.

Wilkins later modified his nomenclature to include various forms of the syndrome under the umbrella designation, “Syndrome of Gonadal Dysgenesis.” He stoutly maintained, however, that this syndrome should never have been designated “Turner Syndrome.”

### The Syndrome of Testicular Feminization

### Androgen Insensitivity Syndrome (AIS)

In his 1950 textbook, Wilkins described another kind of sexual ambiguity. She was an attractive 30 year old woman who had never menstruated and who lacked sexual hair. Surgical exploration of her pelvis revealed absence of uterus, but presence of testes and rudiments of epididymis and vas deferens.

Her urinary 17 ketosteroids ranged between 15 and 20 mg/24 hr and her androgens measured by bioassay were similarly high for a female but normal for a male.

Wilkins treated her with methyl testosterone, up to 50 mg/day with no discernable effect on seborrhea, sexual hair, or clitoral enlargement. He therefore postulated that all of her findings were due to resistance to androgen action at the peripheral level. He called these patients “hairless ladies with testes.” Several other patients were followed later. Their karyotypes were shown to be 46 XY with a plasma testosterone level usually above normal male range. The locus of the gene for the androgen receptor was determined by Drs. Meyer W, Migeon B, and Migeon C. [11] The gene itself was isolated by Drs. DB Lubahn, TR Brown, JA Simental, HN Higgs, CJ Migeon, EM Wilson, and FS French. [12]

The name of testicular feminization was changed to androgen insensitivity syndrome by Money J and Migeon C.

Unfortunately, Wilkins’s observation including the correct pathophysiology of the syndrome, published in 1950 in his textbook, was ignored and the disorder was called Swyer’s Syndrome for a while.

Wilkins made many other seminal contributions to Pediatric Endocrinology during the short time that he was engaged in a full time academic career. Perhaps his single contribution that brought the nascent field of Pediatric Endocrinology into prominence was his poster session at the Sixth International Congress of Pediatrics in Zurich in 1950. This extensive display provided examples of each of the now common endocrine disorders in Pediatrics along with a delineation of diagnostic criteria and pathophysiologic basis, insofar as it was then known. This display was the foundation of his text, Disorders of Endocrine Secretions in Childhood and Adolescence.(13) Many European pediatricians came to Baltimore to study under Wilkins and on returning home, they founded the European Society of Pediatric Endocrinology (ESPE). Although the number of American postdoctoral fellows was not large, they and their fellows have made a very large impact on the development of Pediatric Endocrinology in America. Robert Blizzard and Claude Migeon, who succeeded Wilkins as codirectors of Pediatric Endocrinology at Harriett Lane honored their former chief by establishing an annual symposium in his name, and this symposium evolved into the Lawson Wilkins Pediatric Endocrine Society (LWPES).

The legacy left by Wilkins to the discipline of Pediatric Endocrinology is indeed remarkable for an individual who remained in the private practice of Pediatrics until well after his 50^th^ birthday!

I have often wondered whether Wilkins should be included in the pantheon of great scientists. The word science comes from the Greek work “SCIO” which means “to know.” Wilkins’s major tool was insatiable curiosity – he could not stand not knowing. His curiosity led to great things, and I do not hesitate to classify him amongst the great scientists of all time.”

Table 1 shows Fellows of the Pediatric Endocrine Clinic, 1938-1963 (Figures 1, 2, 3, 4, 5, 6, 7, 8, 9)

**Table 1:** Fellows of the Pediatric Endocrine Clinic, 1938-1963

Associates and Fellows from the United States		
Walter Fleischmann	1938-1946	
Roger A. Lewis	1946-1950	
Robert Klein	1948-1950	
Eugenia Rosemberg	1948-1950	
Lytt I. Gardner	1950-1952	
John F. Crigler, Jr.	1950-1951	
Claude J. Migeon	1950-1952	
Samuel H. Silverman	1951-1952	
John Money	1950-1952	
Alfred M. Bongiovanni	1952-1954	
Walter R. Eberlein	1952-1953	
George W. Clayton	1952-1954	
Melvin M. Grumbach	1953-1955	
Thomas Shepard III	1954-1955	
Judson J. Van Wyk	1953-1955	
Robert M. Blizzard	1955-1957	
H. David Mosier, Jr.	1955-1957	
David W. Smith	1955-1956	
Gerald H. Holman	1956-1958	
Robert S. Stempfel, Jr.	1956-1958	
Orville C. Green	1957-1960	
Gloria Steward	1957	
William W. Cleveland	1958-1960	
Heskel M. Haddad	1958-1959	
Malcolm M. Martin	1958	
Raphael R. David	1958-1961	
Thomas Aceto, Jr.	1960-1962	
Alvro M. Camacho	1960-1962	
Frederic M. Kenny	1960-1962	
Wellington Hung	1960-1962	
James Wright	1961-1964	
Jordan W. Finkelstein	1961-1963	
JoAnne Brasel	1962-1965	
Avinoam Kowarski	1962-1965	
John S. Spaulding	1962-1964	
Fellows from Abroad		
Salvador de Majo	Argentina	1948-1950
Gordon Kennedy	England	1951-1952
Jose Cara	Argentina	1951-1952
Henning Andersen	Denmark	1953
Constantine Papadatos	Greece	1954
Edouard Juillard	Switzerland	1955
John Gerrard	England	1956
Donald J. Delahaye	Canada	1956-1957
Fouad Hamaoui	Lebanon	1957
David Alexander	Scotland	1958-1960
Jean Bertrand	France	1955-1956
Jean-Luc de Gennes	France	1957
Cesar Bergada	Argentina	1959-1961
John P. Eckert	Australia	1959-1961
Enrico Delanto	Mexico	1959
Raphael Rappaport	France	1960-1961
Marija Nikesic	Yugoslavia	1960
Paul Malvaux	Belgium	1961-1962
Bernadette Loras	France	1962-1964
Marco A. Rivarola	Argentina	1963-1967
Dagfinn Aarskog	Norway	1960-1962
Morate Morano	Uruguay	1961-1962

**Figure 1:**
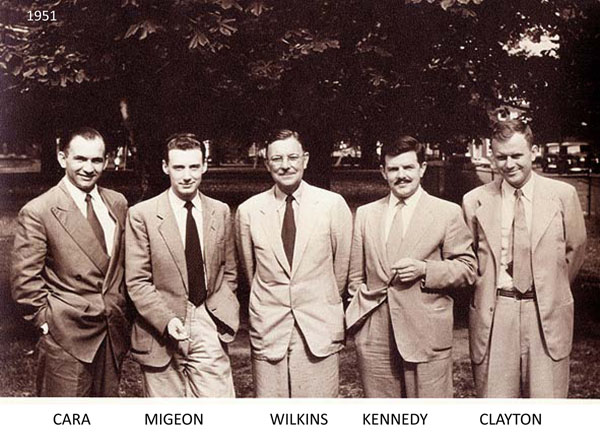
Lawson Wilkins and the Fellows. From left: Jose Cara, Claude Migeon, Lawson Wilkins, Gordon Kennedy, and George Clayton. (1951-1952)

**Figure 2:**
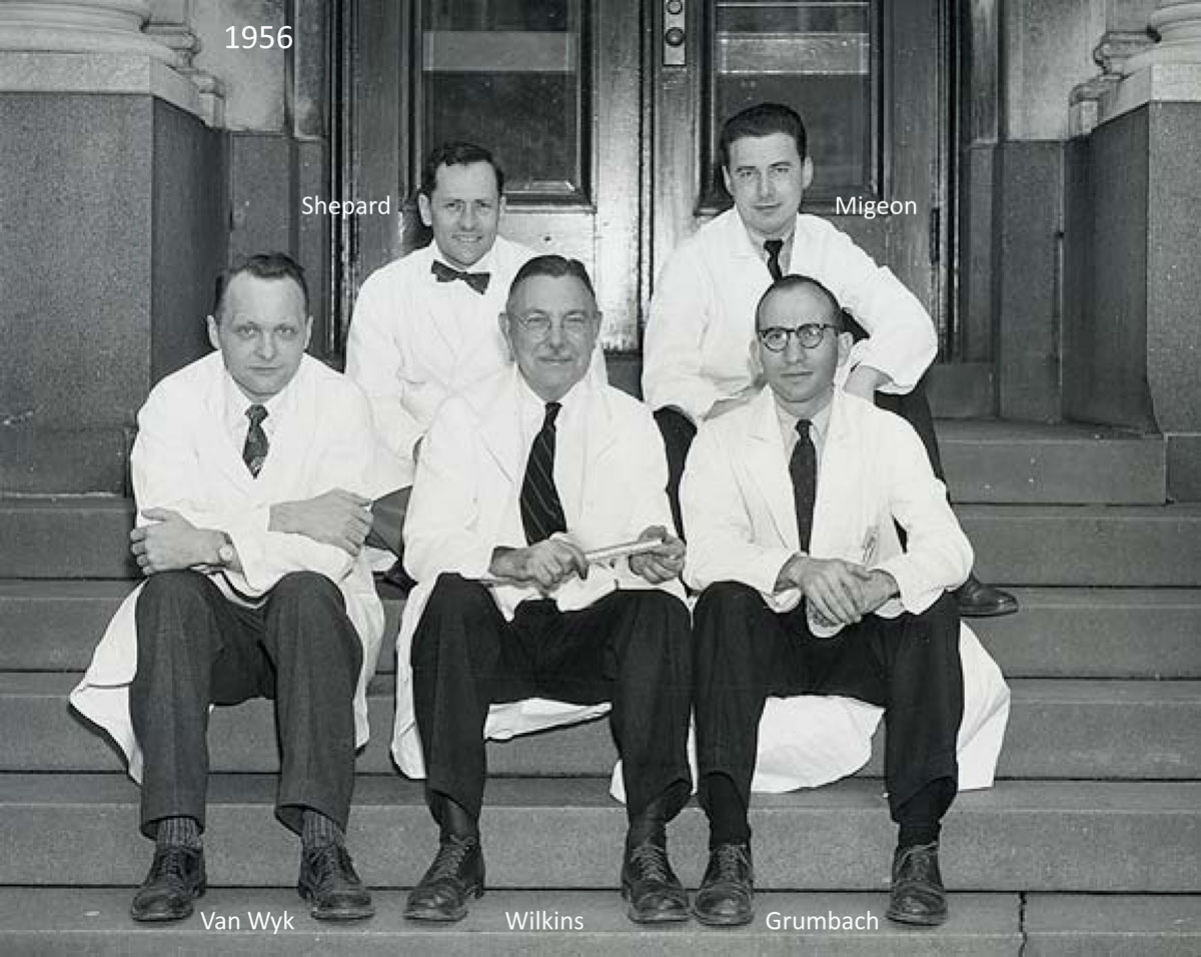
The entrance of the Harriet Lane Home. Front, from left: Judson Van Wyk, Lawson Wilkins, and Melvin Grumbach. Back: Thomas Shepard and Claude Migeon (1955-1956)

**Figure 3:**
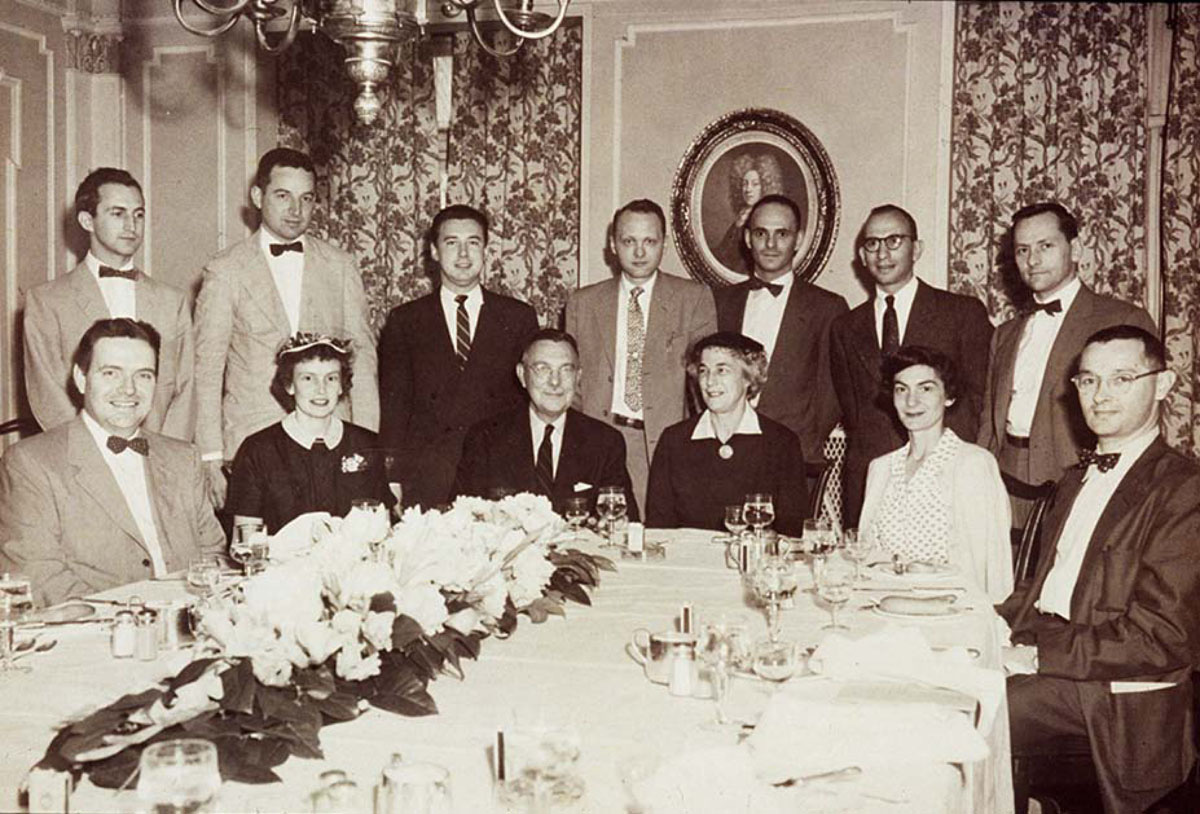
A dinner at Old Point Comfort organized by the fellows in honor of the Wilkins family. Front, from left: Lytt Gardner, Mrs. Crigler, Lawson Wilkins, Mrs. Wilkins, Mrs. Klein, and Robert Klein. Back: Walter Eberlein, John Crigler, Claude Migeon, Judson Van Wyk, Alfred Bongiovanni, Melvin Grumbach, and Thomas Shepard. (1956)

**Figure 4:**
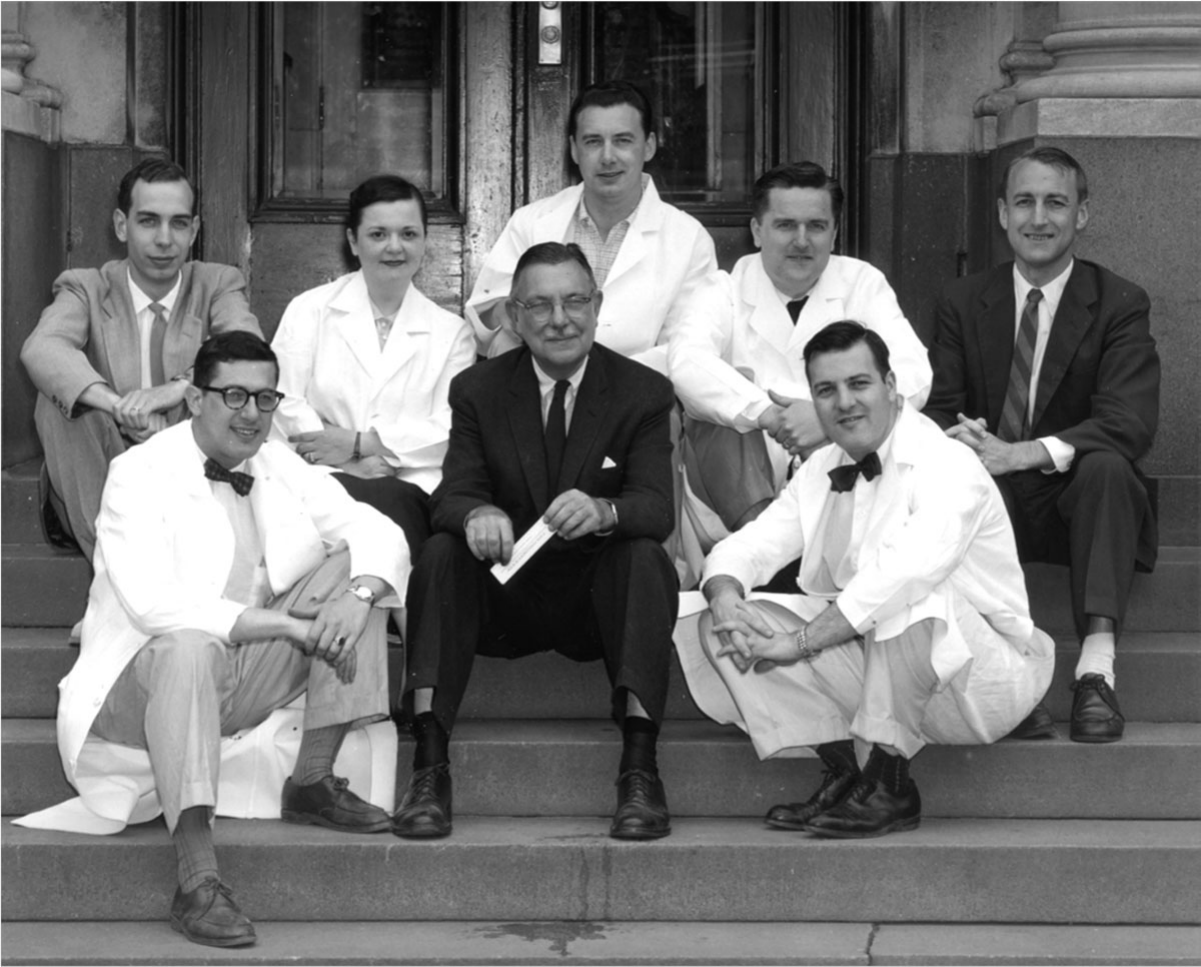
Lawson Wilkins among his fellows, wearing white. From left: Robert Stempfel, Gloria Steward, Claude Migeon, and Gerald Holman, with John Money, far right, of psycho-endocrinology and his fellow, far left. (1957-1958)

**Figure 5:**
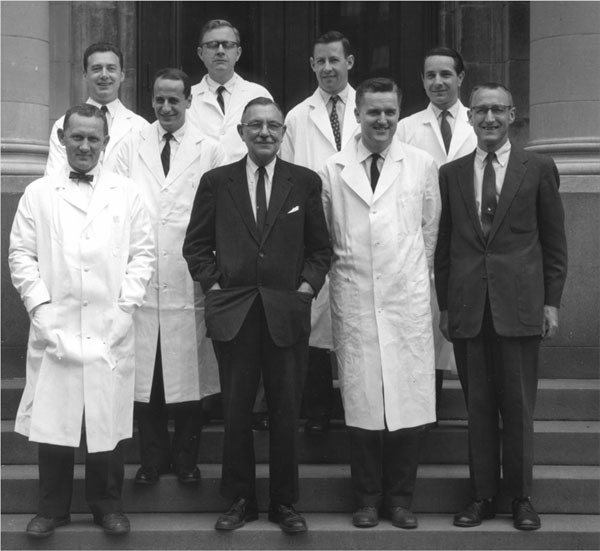
The Pediatric Endocrine Clinic. Front, from left: William Cleveland, Lawson Wilkins, Orville Green, John Money. Back: Claude Migeon, Raphael David, John Eckert, David Alexander, Cesar Bergada. (1959)

**Figure 6:**
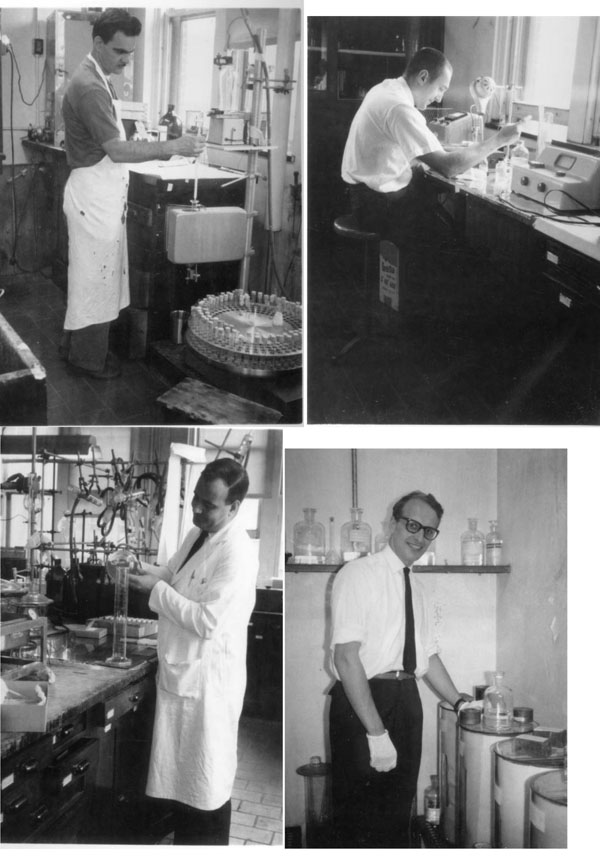
The fellows at work. Top left: Alvro Camacho checking the fraction collector. Top right: Fritz Kenny and the spectrophotometer. Bottom left: James Wright preparing solutions for column chromatography. Bottom right: Paul Malvaux using paper chromatography. (1961-1962)

**Figure 7:**
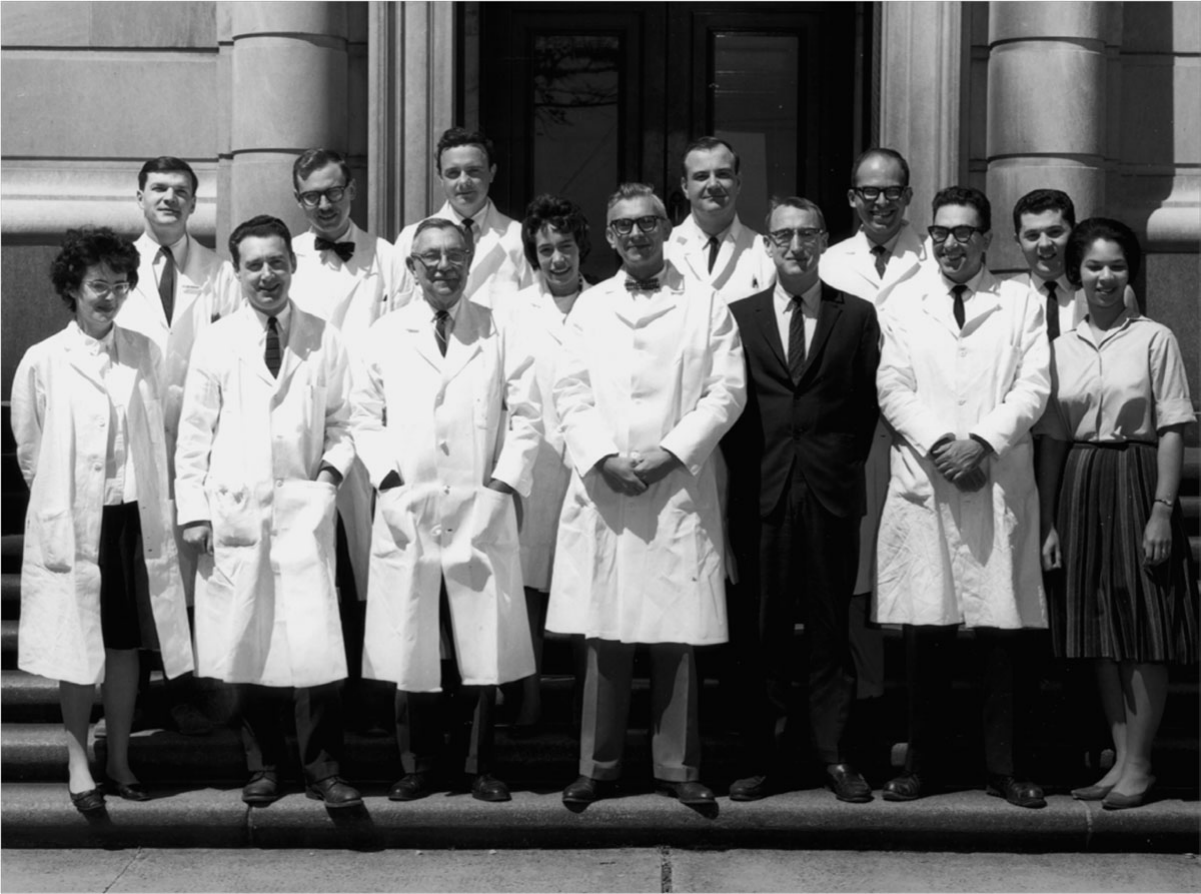
The staff of the Pediatric Endocrine Clinic on the steps of the entrance of Harriet Lane Home. Front, from left: Bernadette Lauras, Claude Migeon, Lawson Wilkins, JoAnne Brasel, Robert Blizzard, John Money, Avinoam Kowarski, and Viola Lewis. Back: John Spaulding, Charles Snipes, Robert Chandler, James Wright, Jordan Finkelstein. (1962-1963)

**Figure 8:**
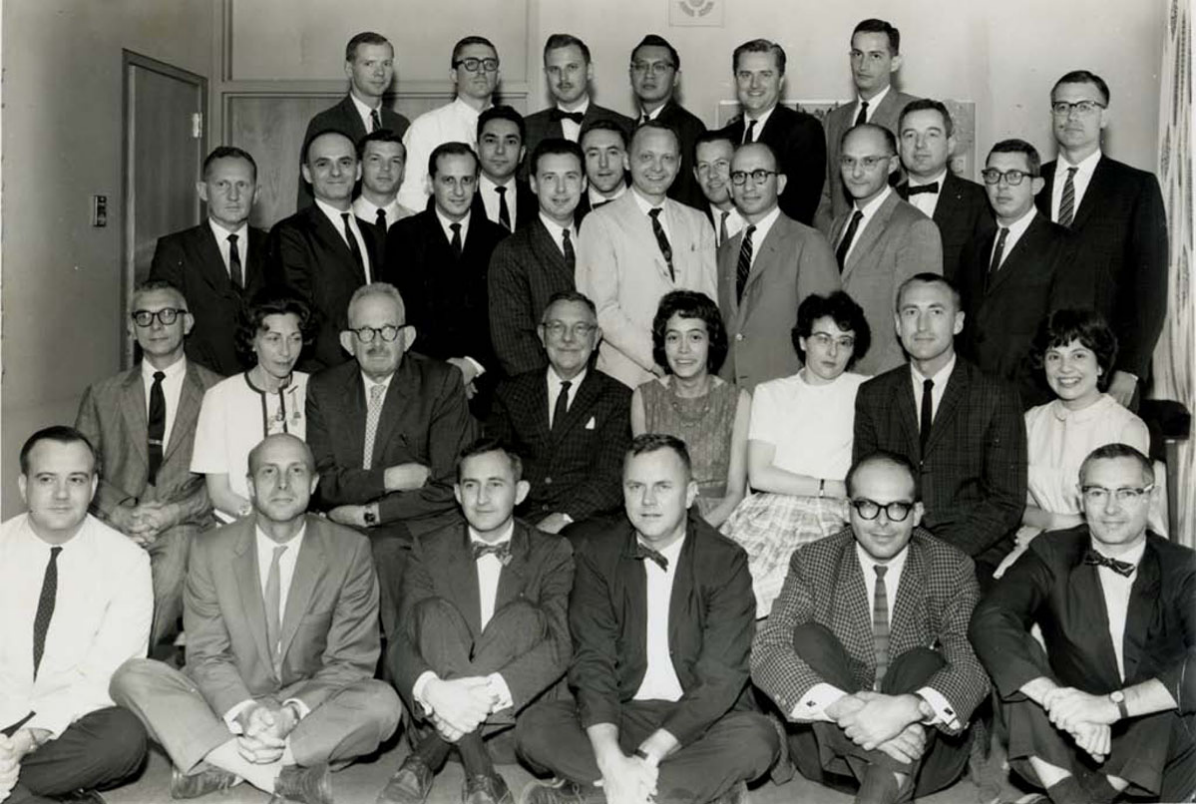
A few months before his death, Lawson Wilkins had called a meeting of his past fellows. Front row, from left: Drs. James Wright, John Gerrard, Walter Eberlein, George Clayton, Jordan Finkelstein, Robert Klein. Second row: Drs. Robert Blizzard, Eugenia Rosemberg, Walter Fleischmann, Lawson Wilkins, JoAnne Brasel, Bernadette Lauras, John Money, Barbara Migeon. Third Row: Drs. William Cleveland, Alfred Bongiovanni, John Spaulding, Raphael David, Avinoam Kowarski, Claude Migeon, Frederic Kenny, Judson Van Wyk, David Alexander, Melvin Grumbach, Malcolm Martin, John Crigler, Robert Stempfel, David Mosier. Fourth Row: Drs. Donald Delahaye, Buford Nichols Jr., Charles Snipes, Wellington Hung, Orville Green, Thomas Aceto Jr. (1963)

**Figure 9:**
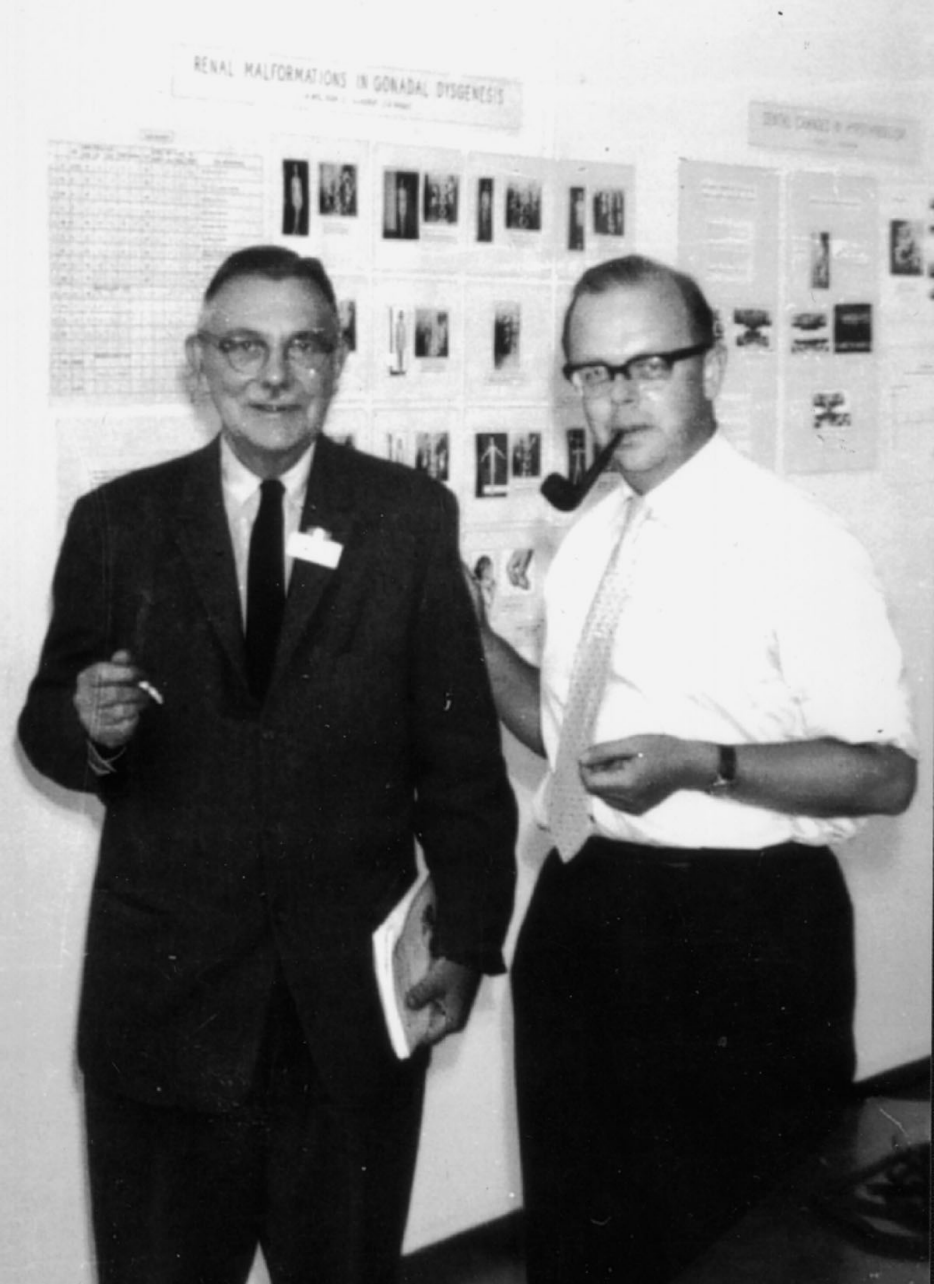
Two great friends: Lawson Wilkins with his eternal cigarette and Henning Anderson with his pipe. (1960)

## Legacy of Lawson Wilkins: the fellows

Dr. Wilkins was not only one of the major creators of the new field of Pediatric Endocrinology but he was also a major mentor of trainees of this field.

Dr. Melvin Grumbach from the University of California in San Francisco analyzed in a sensitive statement what made Dr. Wilkins a great man. He stated the following:

“Lawson was a man for all seasons, a many sided man, clinician, scientist, mentor, educator, encyclopedist, recorder, raconteur, and a pioneer who fathered and fostered the academic and clinical discipline of Pediatric Endocrinology. He cast a large shadow.

Lawson had a restless, inquiring mind, and insatiable curiosity. He was a great clinician but he was also a great scientist – not a bench scientist but a clinical investigator whose laboratory was the clinic, bedside, and operating room. Soon after founding the Endocrine Clinic at Harriet Lane Home, he recruited a series of gifted laboratory directors. They were able to bring to bear the most recent advances in laboratory techniques on the research questions at hand and served as laboratory mentors for a covey of fledgling fellows. Lawson had a profound understanding of the scientific method and the rules of evidence. A keen observer, with an extraordinary ordered mind, meticulous data collector and recorder, he instilled in all of us the importance of attention to detail whether in clinical observations or laboratory work.

Lawson was a hypothesis tester and a critical one. To convince him of the validity of a clinical or laboratory findings or the evidence in favor of particular speculation, one had to do more than one’s homework. A born doubter, his acceptance did not come easily. Parenthetically, he gave little credence to idle speculation or ideas, hypotheses, or notions that were not susceptible to testing.

Once Lawson developed an interest in a clinical problem, he explored it with great determination, commitment, and thought. He had a contagious enthusiasm and the ability to interest others skilled in the laboratory to address clinical problems. He read widely, and freely discussed the clinical issues that interested him with a wide and diverse group of scientists and clinical investigators. He was eminently approachable.

A compassionate man with a lust for life, he was intensely loyal to his fellows, “The Boys,” and took great pride in their achievements. Lawson was an indefatigable worker, but he also loved a good time. Conviviality, get-togethers usually marked by song, and weekends on the Chesapeake were an important part of his and Lu’s life. He was a wonderful informal host. Lawson hated to be alone and thrived on gregariousness.

Who can forget the twinkle in his eyes, the gentle smile, the glowing cigarette held between nicotine-stained fingers, the stentorian resonant voice, his warmth and vigor and the affection which he generated in his fellows?”

Dr. Robert Blizzard, another fellow, wrote a profile of Lawson Wilkins in the Journal of Pediatrics.[14] He had a section on Wilkins as a teacher. He wrote the following:

“Wilkins’ accomplishments as a teacher reached their pinnacle after he joined the faculty at Hopkins in 1946. He taught orally at the bedside, in the clinic, and in the classroom and through the written word in his manuscripts. He documented, through photography and charts, the longitudinal manifestations of endocrine, metabolic, and dysmorphologic diseases; this led to “poster teaching sessions” at the American Academy of Pediatrics annual meetings in the late 1940s and early 1950s. Because of the favorable reception, he published this material in the first edition (1950) of his textbook entitled, *The Diagnosis and Treatment of Endocrine Disorders in Childhood and Adolescence*. His book excelled as a model teaching text because of the clarity of the writing and the case histories which were frequently longitudinal.

The memorable Saturday endocrinology clinics were held in the Harriet Lane Home outpatient department from 9:00am to 1:00pm and were attended by many residents, faculty members, fellows, and students. After the patients were all seen by a fellow or resident, the presentations and discussions began. As Wilkins paced back and forth across the floor in his white coat, with hands behind his back, he discussed what was known about each patient, what was known about the patient’s condition, and what was not known. As should be true of all great teachers, he welcomed thoughts, questions and comments.”

The following listing of the many fellows of Lawson Wilkins and specific comments about these fellows was written in collaboration by William Cleveland and Claude Migeon. Efforts were made to be accurate but we apologize for any error or omission.

**Walter Fleischmann, Ph.D.** (1938-1946) was born and educated in Vienna. His father Carl was an obstetrician, a friend of Sigmund Freud, and he delivered Anne Freud. Walter was a Ph.D. involved in research. In 1938, he was at a conference in Chicago at the time of the Munich Agreement. He was advised to stay in the United States and Lawson Wilkins gave him a job at Johns Hopkins University.

Walter was able to bring his wife and daughter, Ruth, to the US after going to Antwerp. His parents and siblings went to England when they settled. Ruth Fleischmann Weiner wrote, “In effect, Lawson Wilkins saved the lives of the Fleischmann family.”

Walter worked on the effects of various androgenic steroids on creatine metabolism in humans. He was also involved in the attempt to suppress adrenal hypersecretion by various synthetic steroids, unfortunately with effect until cortisone was used. He joined the Veteran Administration Hospital of Baltimore and, later worked in Tennessee.

**Roger Lewis, M.D.** (1946-1950) was involved with the clinic collaborating with colleagues who carried out several studies of thyroid function and started the treatment of children with congenital adrenal hyperplasia.

**Eugenia Rosemberg, M.D.** (1948-1950) came from Argentina and worked with Roger Lewis and Robert Klein, helping the investigation of the patients with congenital adrenal hyperplasia. When she left, she went to the NIH and later joined the steroid group in Shrewsbury, Massachusetts with Dr. Gregory Pincus. Dr. Gregory Pincus and Dr. M.C. Chang were the first to synthesize the combined oral contraceptive pill. Later, Eugenia established her Pediatric Endocrine Clinic at the Worchester Hospital.

**Salvatore de Majo, M.D.** (1948-1950) graduated from medical school in Buenos Aires. He came to learn at the clinic and the treatment of its patients. In addition, he worked with Dr. Lewis on the effects of ovariectomy on albino rats, as well as on the effects of various synthetic steroids on the rat adrenals. When he returned home, he became the director of the Pediatric Endocrine Clinic at the Hospital de Niños in Buenos Aires.

**Robert Z. Klein, M.D.** (1948-1950) graduated from the Harvard Medical School and had training in Pediatrics at the Boston Children’s Hospital. He was an instructor at Johns Hopkins working in Endocrinology with Wilkins and was co-author of some of the pioneering publications about adrenal hyperplasia. He left Hopkins for the University of Pittsburgh where he established a highly regarded program in Endocrinology, continuing research and training fellows. He subsequently held professorships at Boston University and Dartmouth. He continued to make valuable contributions particularly in the area of measuring outcome in congenital hypothyroidism and its relationship to treatment. In this work, he directed the New England Congenital Hypothyroidism Collective.

**Lytt Gardner, M.D.** (1950-1952) was originally from North Carolina and could make the best julep in the world. He directed the laboratories at Hopkins and contributed to the work on congenital adrenal hyperplasia. He established a pediatric endocrine program at the new State University of New York Upstate Medical Center in Syracuse where he remained until his death in 1986. He was succeeded by Dr. Robert A. Richman. Dr. Gardner’s life, professional career, and many valuable contributions have been described by Dr. Mary L. Voorhees (former fellow), and includes a notable list of pediatric endocrinologists trained by Gardner. His work resulted in a highly respected textbook, Endocrine and Genetic Diseases of Childhood and Adolescence.

**John F. Crigler, Jr., M.D.** (1950-1951) was a member of the house staff at Johns Hopkins. Prior to joining the Wilkins group, he was co-author of many of the early papers on treatment of adrenal hyperplasia with cortisone. He then went to Boston Children’s Hospital as a member of the faculty of the Harvard Medical School. He established a very productive endocrinology division. His training program included 70 fellows from all over the world. John remained in this position until his retirement.

**Claude J. Migeon, M.D.** (1950-1952) completed his medical education and pediatric residency in Paris, France. A Fulbright Fellowship brought him to Lawson Wilkins for two years. After three very productive years with Leo T. Samuels in Salt Lake City studying cortisol secretion and metabolism in humans, he returned to Johns Hopkins, and stayed there for the rest of his long career. In 1960, he was named the Co-Director of the Pediatric Endocrine Clinic with Robert Blizzard and was named Director in 1974 until 1994. During these 30 years, he had many fellows from the US and abroad.

**John Money, Ph.D.** (1951) led a group studying the psychologic factors involved in pediatric endocrine disorders. Other valuable contributions to this group came from the John and Joan Hampson. When John Money was asked how he came to Johns Hopkins, he responded as follows:

“In 1949-1950, as a graduate student at Harvard, I had a student appointment at Massachusetts General Hospital, writing a dissertation on the psychosexuality of hermaphroditism. Fuller Albright and Fred Bartter arranged for me to meet Lawson Wilkins when he attended the American Academy of Pediatrics Annual Meeting in Boston and gave his popular workshop on the adrenogenital syndrome of female pseudohermaphroditism. The efficacy of cortisone, newly synthesized for the treatment of the syndrome was simultaneously confirmed at MGH and Johns Hopkins in the first week of 1950.

Lawson and I got along well professionally, perhaps because I had already resolved to use pediatric not psychoanalytic English. He approved my request to visit Hopkins to abstract psychological information from the files of patients whose cases had been published by hospital number, and to interview maybe one or two of them for my dissertation. When the written letter of invitation arrived however, it was to invite me to work in the pediatric endocrine clinic full-time. I began in July 1951.

It has always seemed remarkable to me that this very pragmatic, chart-making man, not much given to psychologizing, should have had the prescience to recognize that he was on to something basically very important psychologically in treating people with hormones.

Although I was not a postdoctoral fellow in Pediatric Endocrinology, Lawson Wilkins provided me, especially at the famed Saturday clinic, with as much Pediatric Endocrinology as I needed to become the first pediatric psychoendocrinologist. Here resides the outstanding feature of his intellectual and academic grandeur as it most personally affected me.

I think that Lawson Wilkins metaphorically adopted all of his trainees, his “boys” (this was not the age of equal opportunity), as substitutes for his only son whom he lost at age 16 in a postal truck auto accident. Double-twisting the helix, he had also himself become a stand in for his son, starting a new career as if on his son’s behalf. Confronting his loss, he gave up general pediatrics and became a full time pediatric endocrinologist. In a sense, he was on an equal footing with his fellows as one of us, not an overbearing father, but a Socratic teacher.”

**Sam Silverman, M.D.** (1951-1952) was interested only in clinical studies. He got involved with the special form of congenital adrenal hyperplasia, the hypertensive form and the salt-losing form. He also studied “Precocious Adrenarche.” He left Baltimore to go into practice at Pediatric Endocrinology in New Jersey.

**Alfred M. Bongiovanni, M.D.** (1952-1955) and **Walter Eberlein, M.D.** (1952-1953) became well known for their contributions to the study of abnormal secretion of adrenal steroids in the various forms of congenital adrenal hyperplasia. They returned to Philadelphia to establish a Division of Pediatric Endocrinology. Eventually, Dr. Bongiovanni became Professor and Chairman at the Department of Pediatrics at the University of Pennsylvania from 1963 to 1972. Following this, he became an itinerant professor holding posts at the University of Ife, Nigeria, Cairo, and the Catholic University of Ponce-de-Leon in Puerto Rico. He later became Dean of this school. He then returned to the faculty of the University of Pennsylvania in 1980 where he remained until his death in 1984.

**George W. Clayton, M.D.** (1952-1954) helped Dr. Bongiovanni as shown by a series of publications. Clayton returned to his beloved Texas where he established a program at Baylor College of Medicine in Houston. He was Professor of Pediatrics and director of the Endocrine Division until his retirement. He moved to Galveston and his principal activities relate to fishing, traveling, and his ongoing interest in the history of the Civil War.

**Melvin M. Grumbach, M.D.** (1953-1955) was busy and very productive. He made a team with Dr. Judson Van Wyk, so their publications were conjoint. The main contributions were related to the study of sex chromosomes in ovarian agenesis and a genetic study of congenital adrenal hyperplasia with Dr. Barton Childs.

He left Baltimore to head a division of Pediatric Endocrinology at Babies Hospital in New York. Among the many fellows he trained, one of the most outstanding was Selna Kaplan. When Dr. Grumbach moved to a position at the University of California at San Francisco, Selna went with him. An outstanding Division has flourished as a result of their efforts. Many leading endocrinologists have been trained in their program.

Mel became chairman of the Department of Pediatrics at UCSF until his retirement. Distinguished colleagues have included Selna, Felix Conte, and Walter L. Miller.

**Judson J. Van Wyk, M.D.** (1953-1955) was one of Wilkins’s most devoted disciples. Jud was involved with Grumbach and Bongiovanni in lab work during his fellowship. He left for the University of North Carolina, where he started a vigorous Clinic of Pediatric Endocrinology. His colleagues at UNC included Frank French, Joseph D’Ercole, and more recently Louis Underwood who assumed direction of the Division. Dr. Van Wyk trained many fellows including Dr. Stockholm. He made many important contributions, particularly in his work relating to somatomedin (IGF-1) as a growth factor.

**Thomas H. Shepard, M.D.** (1954-1955) moved to Seattle after completion of a year with Wilkins where he was appointed to the faculty at the University of Washington. He directed a division of Pediatric Endocrinology at the Children’s Orthopedic Hospital from 1956-1961. He developed an interest in embryology and teratology; studies in this area became the main focus of his career. After a year as a research associate in embryology in the Department of Anatomy, University of Florida College of Medicine, he spent another 6 months in the Fetal Laboratory, University of Copenhagen with Dr. Henning Anderson.

Upon his return to Seattle, he continued his academic career and headed a Birth Defects Research Laboratory for 30 years. He became Emeritus Professor in 1993. He published extensively – some reports involving Endocrinology but most related to embryology and teratology.

**Robert M. Blizzard, M.D.** (1955-1957) was destined to play a major role in the Harriet Lane program and in Pediatric Endocrinology. After his training, he left Hopkins to direct a division at Ohio State in Columbus. Upon Wilkins’s retirement in 1960, Bob was chosen to succeed him as Clinical Director of the Harriet Lane Program with Claude Migeon as Laboratory Director. He was asked to serve as acting Chairman of the Department of Pediatrics at Hopkins following the departure of Dr. Robert Cooke. He did this for 15 months until the position was taken over by Dr. Littlefield.

Later, Bob was recruited to the Chairmanship of the Department of Pediatrics at the University of Virginia in Charlottesville, a position which he held until his retirement. During his tenure as chairman, he maintained a productive career in Endocrinology, working with outstanding colleagues including Alan Rogol and Anne Johanson. In his “retirement,” he remains very productive with many roles including chairmanship of the Board of the Genentech Foundation.

**Gerald H. Holman, M.D.** (1956-1958) became a peripatetic professor after completing his residency and fellowship at Hopkins. He spent 1958-1961 in Saskatoon and 1961-1964 in Kansas City. From 1964-1969, he was Professor and Chairman at the Medical College of Georgia. From 1969-1974, he was Professor and Head of Pediatrics at the University of Calgary. From 1974-1975, he was Professor and Chairman at Eastern Virginia School of Medicine. From 1975-1979, he was Dean at Norfolk. He then went to Amarillo, Texas and held various administrative and faculty positions there, including directing a division of Pediatric Endocrinology. In these various situations, he participated in a prestigious number of programs and committees. His publications are expectedly wide ranging including Endocrinology, nutrition, ethics, and the social aspects of medical care.

**Robert Stempfel, M.D.** (1956-1958) went from the training program to appointment in the Department of Pediatrics, Duke University, School of Medicine as director of Pediatric Endocrinology. He maintained a clinical and laboratory program until his recruitment to the Chairmanship of Pediatrics at the newly established University of California School of Medicine at Davis. His involvement in Endocrinology there was somewhat peripheral. He recruited Dr. Bagher Sheikholislam to his faculty; Bagher continues in the program there. In 1971, Stempfel was enticed to come to Miami as Director of the Mailman Center for Child Development, Professor of Pediatrics and Associate Chairman. He maintained this position until 1995. During his tenure he became heavily involved in the political aspects of child care in the State of Florida and represented the School of Medicine, University of Miami, in Tallahassee in legislative matters. After giving up directorship of the Center he moved from Miami to Key Largo and maintained his political representation until 1995 when he retired completely. He wasable to devote his full attention to his abiding interest in fishing in the Keys and the Bahamas.

**Orville C. Green, M.D.** (1957-1960) left Baltimore to establish a program at the Children’s Memorial Hospital in the Department of Pediatrics, School of Medicine, Northwestern University. He maintained a flourishing Division, productive both in research and training until his retirement. He currently spends winters in Sarasota and summers in Wilmette and Nantucket. Among his trainees was Dr. Robert Winters who has succeeded Orville in the direction of the Division. When asked about Lawson Wilkins, Orville recalled two statements made by Wilkins:

1. “Never take money from a drug company – take their products for experimentation if they seem worthwhile but never take money – they will never get off your back.”

2. “The clinical use of the science of endocrinology is to diagnose and treat conditions of excess and deficiency.” Orville adds, “I think this basic principle should be reaffirmed.”

**Gloria Steward, M.D.** (1957) left the program after 6 months.

**David H. Mosier Jr., M.D.** (1955-1957) was from Kansas. He went to the University of Notre Dame and Johns Hopkins Medical School. After a Pediatric Residency at the University of Southern California, he came back to Hopkins as a fellow in Pediatric Endocrinology with Wilkins. Afterwards, he returned to California, where he was named Professor of Pediatrics at the University of California, Irvine. As head of the Division of Endocrinology, he has been active in experimental and clinical research of the various problems influencing growth.

**David W. Smith, M.D.** (1955-1956) spent two years in the United States Army after completing his residency in Pediatrics. He returned after two years to complete one year of fellowship with Wilkins in Endocrinology. He then spent a year in practice in Los Gatos, California before accepting a faculty appointment at the University of Wisconsin. He established an Endocrinology training program producing third generation fellows including Dr. Arlan Rosenbloom. He then took a sabbatical in Zurich and became interested in genetics. Like his mentor, he then proceeded to pioneer a new field, dysmorphology, and published the definitive text on the subject: “Recognizable Patterns of Human Malformation” which has continued as the authoritative compendium of dysmorphic syndromes. In 1966, he moved to the University of Washington where he remained as Professor of Pediatrics until his untimely death at the age of 55 years. His character and contributions are described in an introduction to a Festschrift issue of the Journal of Pediatrics in 1982.

**William W. Cleveland, M.D.** (1958-1961) left the position of Assistant Professor at the University of Miami to come to Johns Hopkins as fellow with Lawson Wilkins. He worked diligently with patients and wrote several papers. William was also a close friend; he and his wife were our witnesses at my marriage to Barbara in 1960.

Cleveland returned to his faculty position at the fledgling School of Medicine, University of Miami. He continued to direct a Division of Endocrinology for the ensuing 36 years. During twenty of those years (1969-1989), he was also Chairman of the rapidly expanding Department of Pediatrics.

**Malcolm M. Martin, M.D.** (1958-1959) was originally from England. He helped review the question of pituitary dwarfism. He then went to Georgetown University in Washington D.C. where he directed the endocrine clinic.

**Raphael David, M.D.** (1958-1962) was educated in the French system of Cairo. He migrated to the United States and was a Hopkins pediatric resident before joining the clinic of Lawson Wilkins. At the time of Wilkins’s first heart attack, Ralph was his constant companion in the Marburg Building. With time, they grew very friendly.

Ralph has devoted his career to an excellent Division of Endocrinology in the Department of Pediatrics at the School of Medicine at New York University. His program has been highly productive in training and research. As might be expected from his urbane nature, he is a confirmed and enthusiastic New Yorker.

**David Alexander, M.D.** (1958-1960) was originally from Scotland. He did his pediatric residency at Johns Hopkins. After residency, he joined the Pediatric Endocrine Clinic. Following one year of clinical work, he went with his Scottish countryman Dr. Malcolm Ferguson-Smith in cytogenetics. Malcolm was an expert in the investigation of karyotypes in patients. Another notable accomplishment during training of David was to marry Jean, another Hopkins pediatric resident. They moved to Kingston, Ontario from Baltimore where they have maintained practices, David in Endocrinology and Jean in child development.

**Cesar Bergada, M.D.** (1959-1961) and his wife Estela came from prominent families in Buenos Aires. Cesar worked with Dr. Salvador de Majo and Dr. Martin Cullen. Cesar and Estela met Lawson when Lawson and his family visited South America in 1957. As a fellow, Cesar was involved in the study of DSD (intersex) patients in collaboration with Dr. Howard Jones in Gynecology

Upon completion of his training in Baltimore, Cesar returned to Buenos Aires to establish an endocrine division at the Hospital de Niños R. Gutierrez, which he directed for more than three decades. Cesar was recognized as one of the outstanding pediatric endocrinologists in South America. He has trained many endocrinologists, including his own son and Marco Rivarola.

**Thomas Aceto Jr., M.D.** (1960-1962) left the army in 1960 to join the training program at Hopkins, along with Alvro Camacho and Fritz Kenny. After that, he went to the Children’s Hospital of University of Buffalo. Then he became the Chairman of Pediatrics at the University of South Dakota at Sioux Falls. His next promotion was as Medical Director of the Cardinal Glennon Memorial Hospital at St. Louis University. Unfortunately, Thomas developed Alzheimer’s disease and died in 2009.

**Frederic M. Kenny, M.D.** (1960-1962) graduated from Princeton. He attended Hopkins for medical school and pediatric resident training. He married Jean, a colleague in resident training. For two years (1958-1960) he was Pediatrician in Chief at the U.S. Naval Hospital in Annapolis.

In 1960, he returned to Hopkins for two very busy years in Pediatric Endocrinology. Next, he moved to the Children’s Hospital at the University of Pittsburgh. There, he took the place of Dr. Robert Klein and worked at a very large diabetic clinic, which attracted other Hopkins fellows, Dr. Alan Drash and Dr. Dorothy Becker. Unfortunately, Fritz battled with depression and ended his own life in 1978.

**Wellington Hung, M.D.** (1960-1962) did most of his training and spent most of his life in Washington D.C. at American University, the Medical School of George Washington University, and the Children’s Hospital of D.C.

After two years at Hopkins doing clinical research, he returned to Children’s Hospital, National Medical Center, Washington D.C.

During his stay at Hopkins, he had learned about thyroidology from Dr. Robert Blizzard, and he became an expert in this field.

Along with colleagues Dr. August and Dr. Glasgow, he wrote a succinct book of Pediatric Endocrinology.

**Dagfinn Aarskog, M.D.** (1962-1963) did his undergraduate and medical school studies in Norway at the University of Oslo and Bergen, respectively. He did his pediatric training at the University of Bergen prior to coming to Baltimore. Upon his return to Norway, he became Professor and Chairman of the University of Bergen in 1971. In 1984, he was Dean of the Faculty of Medicine. Dagfinn contributed greatly to the field of Pediatric Endocrinology, including describing the Aarskog Syndrome.

**John Spaulding, M.D.** (1962-1963) was a fellow for only one year during which he contributed to the study of patients with the rare problem of unresponsiveness of the adrenals to ACTH. After his fellowship, he rejoined the University of Kansas Medical Center where he directed the clinic of Pediatric Endocrinology.

**JoAnne Brasel, M.D.** (1962-1965) was a lovely lady who lived her life fighting a slow progressive, disabling neurological problem. She went to medical school at the University of Colorado and did her residency at Cornell University.

From 1962 to 1965, she joined the Hopkins Pediatric Endocrinology program and joined the staff in 1968. She became interested in growth and development research, working successively at Cornell, Columbia, and Harbor-UCLA Medical Center. She was in charge of the training program at Harbor-UCLA. When she died in 2007, we lost a great scientist and a wonderful friend.

**Jordan Finkelstein, M.D.** (1961-1963) spent two years at Hopkins. He worked with Dr. Kowarski on the determination of aldosterone secretion in rats in various conditions. Particularly, the study of patients with congenital adrenal hyperplasia showed that all of them have a salt-losing tendency, but those who had a milder mutation of their 21-hydroxylase gene could compensate with increased aldosterone secretion, therefore avoiding salt-loss. In contrast, the patients with severe mutations could not compensate and were salt-losers. Jordan returned to Montefiore Hospital in the Bronx.

**A. Avinoam Kowarski, M.D.** (1962-1965) was born in Tel Aviv, Israel. He started medical school in Lausanne, Switzerland and finished at the Hebrew University of Jerusalem. After three years as fellow at Hopkins and two years at Hadassah University Hospital, he returned to the pediatric endocrine clinic, where he was involved with laboratory research. In 1981, he opened the Pediatric Endocrine Clinic at the University of Maryland.

**Gordon Kennedy, M.D.** (1951-1952) came from Cambridge, England, with the purpose to see the function of the Pediatric Endocrine Clinic. He was an expert on experimental brain lesions to rats which resulted in increased appetite and gross obesity.

**José Cara, M.D.** (1951-1952) came from Argentina. He was interested in clinical work and eventually joined Lytt Gardner at Syracuse. José’s son was also an endocrinologist.

Other doctors from abroad were truly involved in all the activities of the Clinic. The first was **Dr. Jean Bertrand** (1955-1956) from Lyon, France. We became close friends and after his return home, he went to join me in Stockholm to help me at the Karolinska Hospital where we worked with Dr. Carl Gemzell on the transplacental passage of steroids. **Bernadette Loras, M.D.** (1962-1964), **Maguelone Forest, M.D.**, and **José Saez, M.D.** were three pupils of Jean Bertrand who worked in his laboratories.

**Raphael Rappaport, M.D.** (1960-1961) came from the Hopital de Enfants Malades and then returned home to create an important clinic. **Paul Malvaux, M.D.** (1961-1962) came from Louvain and similarly returned to his institution to be the Director of Pediatric Endocrinology. Both of them returned to Johns Hopkins to help with the clinical load when Dr. Blizzard was temporary Chief of Pediatrics.

Some doctors who had not been fellows felt that Wilkins was their mentor. For example, **Andrea Prader** of Zurich, Switzerland, the founder of the European Society of Pediatric Endocrinology, wrote the following:

“Because Lawson had the greatest impact on my training as a clinician and clinical investigator, I add a personal note regarding Lawson. He, along with Guido Fanconi, stimulated my interests in endocrinology, metabolism, genetics, and growth. Both were superb clinicians and forceful and enthusiastic teachers.

Their scientific contributions were not the results of systematic laboratory research in the framework of a sophisticated research program, but the offshoots of very careful clinical observations combined with a deep interest in the patient and his problems. They used charts to follow the courses of diseases and the growth of patients. They recognized important disease aspects not noted by others, were able to add new insights into many diseases, and offered challenging new speculations.”

This was the same for Henning Anderson of Denmark. Henning had a special relationship with Lawson. When they were together, Lawson and Henning considered themselves as Vikings and Harold the Blue Tooth was one of their ancestors.

Another important admirer of Lawson Wilkins was Professor Alfred Jost, head of the Laboratory of Comparative Physiology, Faculty of Sciences at the University of Paris. After Wilkins’s death in 1963, Dr. Jost gave the fourth Lawson Wilkins Memorial Lecture on April 26, 1971. On that occasion, he wrote about meeting Lawson Wilkins:

“On my return from Mexico, I visited several eminent American experts in the field on sex differentiation in animals. The first stop was in Baltimore, where I was to meet world-famous scientists at the Department of Biology of The Johns Hopkins University and at the Department of Embryology of the Carnegie Institution of Washington. Dr. Robert K. Burns an Associate in Embryology was very friendly to me during this visit, and he suggested that I should meet at The Johns Hopkins Hospital a clinician by the name of Dr. Lawson Wilkins, who was interested in problems of human genital anomalies. Dr. Burns made the appointment for the early afternoon, and thus I was introduced to Lawson Wilkins. He was 55, I was 33; he was a well-known clinician, I was not even a doctor of medicine. He calmly and patiently followed my description of the rabbit experiments, looking at the rabbit pictures, asked many pertinent questions, and listened to the interpretations proposed for human anomalies. Then he submitted me to a keen clinical examination. I had to comment on the illustrations and reports concerning clinical cases. I thus had the privilege of being among the very first ones who saw the cases later to appear in the first edition of “The Diagnosis and Treatment of Endocrine Disorders in Childhood and Adolescence.” But not only did I have to look at pictures, I was asked for interpretations which were coldly evaluated and screened. Also, I was introduced to extremely important clinical experiments, for instance, those concerning the absence of sensitivity to androgens of “hairless women with testes.”

Several of the fellows from abroad attended the daily clinics of Lawson Wilkins, including **Constantine Papadatos, M.D.** (1955) from Athens, Greece, **Edouard Juillard, M.D.** (1955) from Lausanne, Switzerland, **John Gerrard, M.D.** (1956) and **Donald Delahaye, M.D.** (1956) from Canada, **Fouad Hamaoui, M.D.** (1956) from Beirut, Lebanon, **Jean-Luc de Gennes, M.D.** (1957) from Paris, France, **Enrico Delanto, M.D.** (1959) from Mexico, **Marija Nikesic, M.D.** (1960) from Belgrade, Yugoslavia, **John Eckert, M.D.** (1961) from Australia, and **Morato Marano, M.D.** (1962) from Montevideo, Uruguay.

A bibliography can be found in Additional file 1.

## Conclusion

In 2003, on the 22^nd^ of December, I discovered that I was 80 years old. I was amazed and a little afraid. It had been 50 years since Dr. Wilkins died. I wrote a little note; a copy of which follows:

How is it possible? Already eighty? Pas Possible! So many years. But what can I do if it is true? Resign myself to it hope for another day and be thankful for my past youth

**Figure 10:**
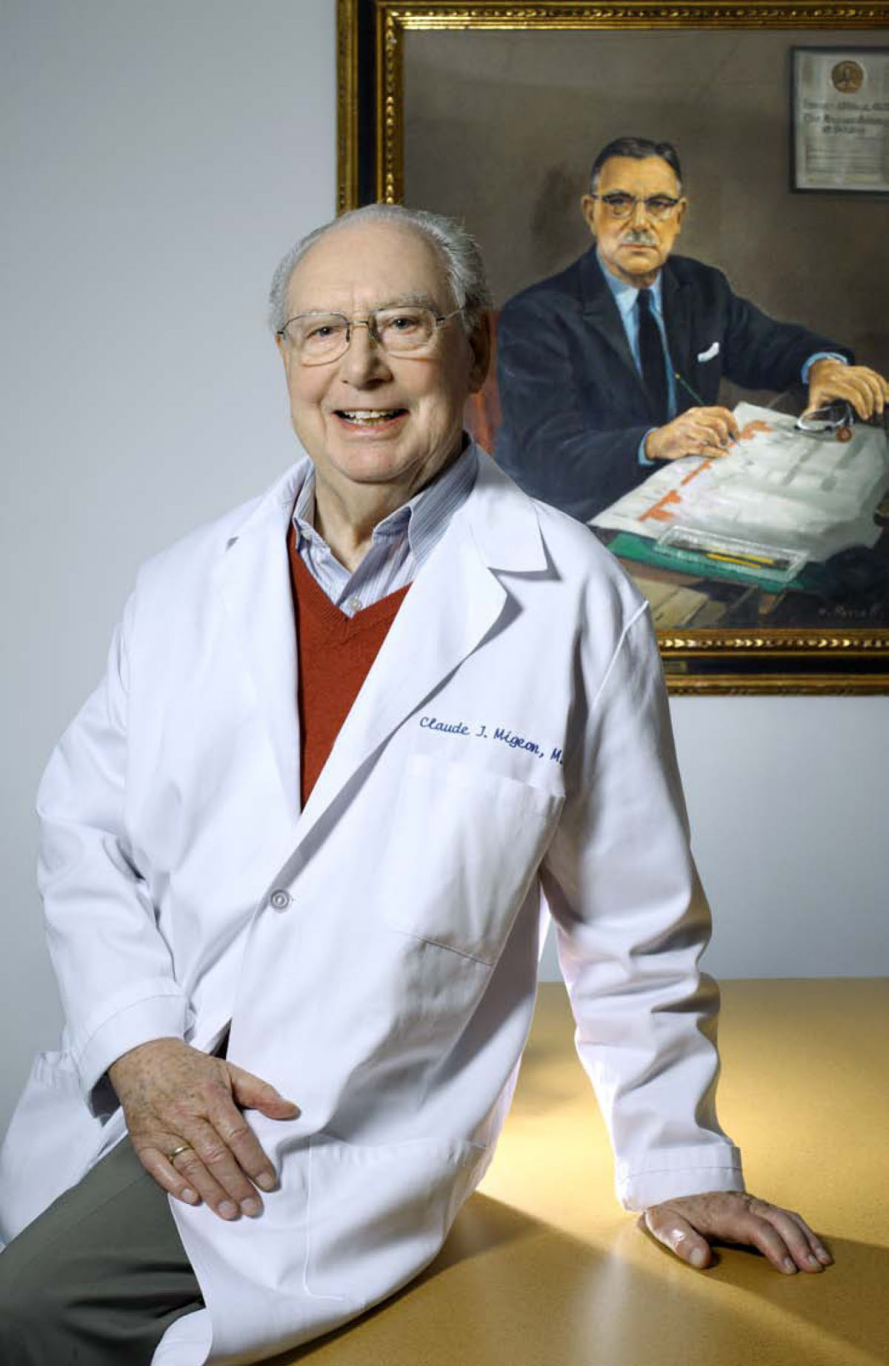
Claude Migeon, M.D. with the famous portrait of Lawson Wilkins, M.D. (November 2011)

Now in 2013, we are ten years later and I am 90 years old (Figure 10). What happened during that extra time? Well, I plan to answer that. Betsy McMaster and I have finished our memoir of Lawson Wilkins.

It is of interest to consider what role “chance” plays for better or worse in life. In 1950, in Paris when I got my MD degree, I was not ready for settling down: get married, open an office of pediatrics, probably in Reims and be a respectful citizen.

Chance permitted me to apply for a Fulbright Fellowship to go to a hospital in Chicago and see my good friends Bill and Stella Nanos. Instead, chance had it that Dr. Wilkins asked for me at Hopkins. At the end of the two year fellowship, Dr. Wilkins made it possible for me to continue working in Salt Lake City. And after 3 years in Utah, he brought me back to Baltimore for the rest of my life.

I want to conclude this memoir in thanking with all of my heart, Dr. Wilkins, for permitting me to have a wonderful life, with my wife Barbara, my children Jacques, Jean-Paul, and Nicole; my many fellows who were like children; my kind colleagues, and all of my good friends.

## Competing interest

The author has no competing interests to disclose.

## Supplementary Material

Additional file 1 bibliography Click here for file

## Declarations

This article has been published as part of *International Journal of Pediatric Endocrinology* Volume 2014 Supplement 1, 2014: Remembering Doctor Lawson Wilkins: a pioneer of pediatric endocrinology. The full contents of the supplement are available online at http://www.ijpeonline.com/supplements/2014/S1.
