# Supplementary material for: Lawson Wilkins and my life: part 3
Source: Int J Pediatr Endocrinol. 2014 May 28;2014(Suppl 1):S4. doi: 10.1186/1687-9856-2014-S1-S4 (PMC4080310; doi:10.1186/1687-9856-2014-S1-S4)
Supplement: Additional file 1 — bibliography [file 1687-9856-2014-S1-S4-S1.docx]

**BIBLIOGRAPHY**

1938

1. **Wilkins, L.** The rates of growth, osseous development, and mental development in cretine as a guide to thyroid treatment. J. Pediat. 12: 429, 1938.

1939

2. **Wilkins, L.** Some problems and methods of diagnosis of cretinism and juvenile hypothyroidism. Delaware State Medical Journal, June, 1939.

1940

3. **Wilkins, L., Fleischmann, W. and Howard, J. E.** Macrogenitosomia precox associated with hyperplasia of the endrogenic tissue of the adrenal and death from the corticoadrenal insufficiency. Endocrinology 26: 385, 1940.

4. **Wilkins, L., Richter, C. P.** A great craving for salt by a child with corticoadrenal insufficiency. J.A.M.A. 114: 866, 1940.

5. **Wilkins, L.** Thyroid medication during childhood. J.A.M.A. 114: 2382, 1940.

6. **Fleischmann, W., Shumacher, H. B., Jr. and Wilkins, L.** The effect of thyroidectomy on serum cholesterol and basal metabolic rate in the rabbit. Am. J. Physiol. 131: 317, 1940.

1941

7. **Wilkins, L.** Epiphysial dysgenesis associated with hypothyroidism. Am. J. Dis. Child. 61: 13, 1941.

8. **Wilkins, L., Fleischmann, W. and Block, W.** Studies on hypothyroidism in childhood: (1) The basal metabolic rate, serum cholesterol and urinary creatine before treatment. J. Clin. Endocrinol. 1: 3, 1941.

9. **Wilkins, L., Fleischmann, W. and Block, W**. Studies on hypothyroidism in childhood: (2) Sensitivity to thyroid medication as measured by the serum cholesterol and the creatine excretion. J. Clin. Endocrinol. 1: 14, 1941.

10. **Wilkins, L. and Fleischmann, W.** Studies on hypothyroidism in childhood: (3) The effect of withdrawal of thyroid therapy upon the serum cholesterol. Relationship of cholesterol, basal metabolic rate, weight and clinical symptoms. J. Clin. Endocrinol. 1: 91, 1941.

11. **Wilkins, L. and Fleischmann, W.** Studies on hypothyroidism in childhood: (4) The creatine and cholesterol response to thyrotropic hormone. J. Clin. Endocrinol. 1: 98, 1941.

12. **Hartman, Carl G. and Fleischmann, W.** Serum cholesterol in the rhesus monkey. Endocrinology 29: 793, 1941.

13. **Wilkins, L.** Recent studies on the diagnosis of hypothyroidism in children. The Pennsylvania Medical Journal 44: 429, 1941.

14. **Wilkins, L. and Fleischmann, W.** The diagnosis of hypothyroidism in childhood. J.A.M.A. 116: 2459, 1941.

15. **Fleischmann, W. and Wilkins, L.** Sterol balance in hypothyroidism. J. Clin. Endocrinol. 1: 799, 1941.

16. **Wilkins, L., Fleischmann, W. and Howard, J. E.** Crestinuris induced by methyltestosterone in the treatment of dwarfed boys and girls. Bull. Johns Hopkins Hosp. 69: 493, 1941.

1942

17. **Fleischmann, W. and Shumacher, H. B., Jr.** The relationship between serum cholesterol and total body cholesterol in experimental hyper-and hypo- thyroidism. Bull. Johns Hopkins Hosp. 71: 175, 1942.

18. **Howard, J.E., Wilkins, L. and Fleischmann, W.** The metabolic and growth effects of various androgens in sexually immature dwarfs. Trans. Assn. Am. Physicians. 57: 212, 1942.

1943

19. **Fleischmann, W., Shumacher, H. B., Jr. and Straus, W. L., Jr.** Influence of age on the effect of thyroidectomy in the rhesus monkey. Endocrinology 32: 238, 1943.

1944

20. **Wilkins, L. and Fleischmann, W.** Sexual infantilism in the female: causes, diagnosis and treatment. J. Clin. Endocrinol. 4: 306, 1944.

21. **Wilkins, L. and Fleischmann, W.** Ovarian agenesis, pathology, associated clinical symptoms and the bearing on the theories of sex differentiation. J. Clin. Endocrinol. 4: 357, 1944.

22. **Frame, E. G., Fleischmann, W. and Wilkins, L.** The influence of a number of endrogenic steroids on the urinary excretion of neutral 17-ketosteroids. Bull. Johns Hopkins Hosp. 75: 95, 1944.

22a. **Fleischman, W. and Fried, Ilse E.** Studies on the hypercholesterolemia of immature fowl induced by estrogen. Fed. Proc., Vol. 3. No. 1, March, 1944.

1945

23. **Wilkins, L. and Fleischmann, W.** Studies on the creatinuris due to methylated steroids. J. Clin. Invest. 24: 21, 1945.

24. **Fleischmann, W. and Fried, I. E.** Studies on the mechanism of the hypercholesterolemis and hypercalcemia induced by estrogen in immature chicks. Endocrinology 36: 406, 1945.

1946

24a. **Fleischmann, W.** Effect of thyroxin on estrogen-induced changes in fowl. Fed. Proc., Vol. 5, No. 1, March, 1946.

25. **Wilkins, L. and Fleischmann, W.** Effects of thyroid on creatine metabolism with a discussion of the mechanism of storage and excretion of creatine bodies. J. Clin. Investigation 25: 360, 1946.

26. **Wilkins, L.** The influence of various androgenic steroids on nitrogen balance and growth. J. Clin. Endocrinol. 6: 383, 1946.

1947

27. **Fleischmann, W. and Breckler, I. A. With the technical assistance of Fisk, A. J.** Mitotic and wound healing activities of the corneal epithelium in thiouracel treated and thyroidectomized rats. Endocrinology 41: 266, 1947.

27a. **Buschke, W., Friedenwald, J. S. and Fleischmann, W.** Studies on the mitotic activity of the corneal epithelium. Methods. The effects of colchicine, ether, cocaine and ephedrin. Bull. Johns Hopkins Hosp. 73: 143, 1943.

1948

28. **Wilkins, L.** Genetic and endocrine factors in the growth and development of childhood and adolescence. Rec. Prog. Horm. Res. 2: 391, 1948.

29. **Wilkins, L**. A feminizing adrenal tumor causing gynecomastia in a boy of five years contrasted with a virilising tumor in a five year old girl. Classification of 70 cases of adrenal tumor in children according to their hormonal manifestations and a review of 11 cases of feminising adrenal tumor in adults. J. Clin. Endocrinol. 8: 111, 1948.

30. **Wilkins, L**. Abnormalities and variations of sexual development during child hood and adolescence. Advances in Pediatrics 3: 159, 1948.

31. **Wilkins, L. and Lewis, R. A.** The renal excretion of steroid hormones in pseudohermaphroditism and male sexual precocity associated with symptoms of Addison's Disease. Trans. Conf. on Metabolic Aspects of Convalescence 17: 210, 1948.

32. **Wilkins, L. and Lewis, R. A.** The relationship of dosage and duration of treatment to adrenal atrophy caused by injection of steroid hormone. Trans. Conf. on Metabolic Aspects of Convalescence 17: 168, 1948.

33. **Gantt, W. H. and Fleischmann, W.** Effect of thyroid therapy on the conditional reflex function in hypothyroidism. Am. J. Psychiatry 104: 673, 1948.

34. **Nyda, M. J., de Majo, S. and Lewis, R. A.** The effect of ovariectomy and physiologic doses of estradiol upon body weight, linear growth and fat content of the female albine rat. Bull. Johns Hopkins Hosp. 83: 279, 1948.

1949

35. **Lewis, R. A. and Wilkins, L.** The effect of adrenocorticotropic hormone in congenital adrenal hyperplasia with virilism and in Cushing's Syndrome treated with methyltestosterone. J. Clin. Invest. 28: 394, 1949.

36. **Hardy, J. and Wilkins, L.** Methyl-testosterone in the treatment of premature infants. J. Pediat. 34: 349, 1949.

37. **Wilkins, L.** Hyperadrenocorticism. Pediatrics 3: 533, 1949.

38. **Lewis, R. A. and Wilkins, L.** The effects of 17-vinyl-testosterone and of other steroids upon the size and cholesterol content of the rat adrenal. Trans. Conf. on Metabolic Interrelations 1: 159, 1949.

39. **Wilkins, L. and Lewis, R. A.** Metabolic effects of ACTH in cases of congenital adrenal hyperplasia of the adrenogenital type and in a case of Cushing's Syndrome. Trans. Conf. on Metabolic Interrelations 1: 149, 1949.

40. **Lewis, R. A., de Majo, S. and Rosenberg, E.** The effects of 17-vinyl-testosterone upon the rat adrenal. Endocrinology 45: 564, 1949.

1950

41. **Wilkins, L., Lewis, R. A. and Klein, R.** The response to ACTH in various types of adrenal hyperplasia. Trans. Armour Laboratorities Conf. on ACTH.

42. **Wilkins, L., Lewis, R. A., Klein, R. and Rosenberg, E.** Die wirkung von cortison auf die ausscheidung der 17-ketosteroids and anderer steroids bei patienten mit kongenitaler nebennierenhyperplasia. Halvetica Paediatrica Acta 5: 418, 1950.

43. **Wilkins, L.** Nebennierenrinden-erkrankungen bein kinde. Sonderabdruck aus der Schweiserischen Medizinischen Wochenschrift. 80. Jahrgang 1950, Nr. 29, Seite 766.

44. **Wilkins, L**. Hypothyroidism in children. Prog. in Clin. Endocrinology, January, 1950.

45. **Rosenberg, E. and Lewis, R. A.** Reducation in eosinophil level of adrenalectomised mice following injection of adrenocorticotropin, 11-dehydre-17-hydroxycorticosterene and lipid extracts of human urine. J. Applied Physiology 3: 164, 1950.

46. **Penneys, R., Thomas, C.B. and Lewis, R. A.** Reduction in the number of circulating eosinophils following induced anoxemia. Bull. Johns Hopkins Hosp. 86: 102, 1950.

47. **Lewis, R. A., Klein, R. and Wiklins, L.** Effect of pituitary growth hormone in dwarfism with osseous retardation and hypoglycemia in a cretin treated with thyroid. J. Clin. Invest. 29: 460, 1950.

48. **Klein, R. and Hanson, J.** Adrenocortical function in the new-born infant as measured by adrenocorticotropic hormone-eosinophil response. Pediatrics 6: 192, 1950.

49. **Lewis, R. A., Klein, R. and Wilkins, L.** Congenital adrenal hyperplasia with pseudohermaphroditism and symptoms of Addison's disease; clinical course following bilateral total adrenalectomy with metabolic studies, pathological findings and discussion of etiology. J. Clin. Endocrinol. 10: 703, 1950.

50. **Schoenbach, E. B., Colsky, J. and Lewis, R. A.** Steroid excretion in patients receiving anti-folic acid compounds. Cancer 3: 844, 1950.

51. **Wilkins, L.** Round Table Discussion of Endocrine Therapy in Childhood. Pediatrics 6: 908, 1950.

52. **Wilkins, L., Lewis, R. A., Klein, R. and Rosenberg, E.** The suppression of androgen secretion by cortisone in a case of congenital adrenal hperplasia. Bull. Johns Hopkins Hosp. 86: 249, 1950.

53. **Lewis, R. A. and Rosenberg, E.** The effects of 17-hydroxy-11-dehydrocorticosterone upon the adrenals of normal and hypophysectomized rats maintained with adrenocorticotropin. Endocrinology 47: 414, 1950.

54. **Klein, R. and Livingstone, S.** The effect of adrenocorticotropic hormone in epilepsy. J. Pediat. 37: 733, 1950.

1951

55. **Klein, R.** Adrenocortical control of sodium and potassium excretion in the newborn. J. Clin. Invest. 30: 318, 1951.

56. **Wilkins, Lawson and (by invitation) Gardner, Lytt I., Crigler, John F., Jr. and Migeon, Claude J.** Treatment of congenital adrenal hyperplasia with cortisone. Trans. Assn. Am. Physicians 64: 160, 1951.

57. **Gardner, L. I., Crigler, J. F., Jr. and Migeon, C. J.** The inhibition of urinary 17-ketosteroid excretion produced by "Benemid". Proc. Soc. Ex. Biol. & Med. 78: 460, 1951.

58. **Wilkins, L., Lewis, R. A., Klein, R., Gardner, L. I., Crigler, J. F., Jr., Rosemberg, E. and Migeon, C. J.** Treatment of congenital adrenal hyperplasia with cortisone. J. Clin. Endocrocrinol. 11: 1, 1951.

59. **Gardner, L. I. and Migeon, C. J.** Le diagnostic des tumeurs virilisantes du cortex surrenalien: effet de la cortisone sur les steroides urinatres et utilisantion d'une methode colorimetrique pour le dosage de la dehydroisoandrosterone. Halvetica Paediatrica Acta 6: 465, 1951

1952

60. **Wilkins, L., Gardner, L. I., Crigler, J. F., Jr., Silverman, S. H. and Migeon, C.J.** Further studies on the treatment of congenital adrenal hyperplasia with cortisone. I. Comparison of oral and intramuscular administration of cortisone, with a note on the suppressive action of comounds F and B on the adrenal. J. Clin. Endocrinol. & Metab. 12: 257, 1952.

61. **Ibid: II.** The effects of cortisone on sexual and somatic development, with an hypothesis concerning the mechanism of feminisation. J. Clin. Endocrinol. & Metab. 12: 277, 1952.

62. **Wilkins, L., Crigler, J. F., Jr., Silverman, S. H., Gardner, L. I. and Migeon, C. J.** Further studies on the treatment of congenital adrenal hyperplasia with cortisone. III. The control of hypartension with cortisone, with a discussion of variations in the type of congenital adrenal hyperplasia and report of a case with probable defect of carbohydrate-regulating hormones. J. Clin. Endocrinol. & Metab. 12: 1015, 1952

63. **Crigler, J.F., Jr., Silverman, S.H. and Wilkins, L.** Further studies on the treatment of congenital adrenal hyperplasia with cortisone. IV. The effect of cortisone and compound *B* in infants with disturbed electrolyte metabolism. Pediatrics 10: 397, 1952.

64. **Wilkins, L. and Ravitch, N.** Adrenocortical tumor arising in liver of 3 year old boy with signs of virilism and Cushing's Syndrome. Pediatrics 9: 671, 1952.

65. **Silverman, S. H., Migeon, C. J., Rosenberg, E. and Wilkins, L.** Precocious growth of sexual hair without other secondary sexual development. "Premature Pubarche" a constitutional variation of adolescence. Pediatrics 10: 426, 1952.

66. **Gardner, L. I., Migeon, C. J., Crigler, J.F., Jr., Silverman, S. H. and Wilkins, L.** Urinary dehydroisoandrosterone in hyperadrenocorticism. J. Clin. Endocrinol. & Metab. 12: 1117, 1952.

67. **Migeon, C. J. and Gardner, L. I.** Urinary estrogens (measured fluorometrically and biologically) in hyperadrenocorticism: influence of cortisone, compound F, compound B and ACTH. J. Clin. Endocrinol. & Metab. 12: 1513, 1952.

68. **Migeon, C. J., Gardner, L. I., Crigler, J. F., Jr. and Wilkins, L.** Effect of cortisone treatment for 28 days on radio-iodine metabolism in normal rats and adrenalectomised rats maintained with desoxycorticosterone. Endocrinology 51: 117, 1952.

69. **Migeon, C. J.** Effect of cortisone on lipids of serum, liver and testes in intact and adrenalectomised rats. Proc. Soc. Exper. Biol. Med. 80: 571, 1952.

70. **Wilkins, L.** Constitutional variations of adolescent development. The Mississippi Doctor. Sept., 1952, p. 107.

71. **Wilkins, L**. The diagnosis of the adrenogenital syndrome and its treatment with cortisone. J. Pediat. 41: 860, 1952.

1953

72. **Migeon, C. J.** Fractionation by countercurrent distribution of urinary estrogens in normal individuals and in patients with hyperadrenocorticism. J. Clin. Endocrinol. & Metab. 13: 674, 1953.

73. **Wilkins, L.** Disturbance in growth. Bull. N. Y. Acad. Med. 29: 280, 1953.

73a. W**ilkins, L**. Dwarfism in Current Therapy.

73b. **Wilkins, L**. Abnormalities of sexual development in children and adolescents "Seminar" of Sharp & Dohms, Oct., 1952.

73c. **Fleischmann, W.** Zur hormonal therapie des hypophysaren infantilisms beim Weibe-Mitterlung eines durch strumn lymphomatose Komplizierten Felles. Wiener Klin. Wchschr. 35: 630, 1952.

74. **Bongiovanni, A. M.** The detection of pregnandiol and pregnantrial in the urine of patients with adrenal hyperplasia. Suppression with cortisone. Bull. Johns Hopkins Hosp. 92: 244, 1953.

1954

75. **Bongiobanni, A. M., Eberlein, W. R and Cara, Jose.** Studies on the metabolism of adrenal steroids in the adrenogenital syndrome. J. Clin. Endocrinol. & Metab. 14: 409, 1954.

76. **Eberlein, W. R.** Aminoaciduris in childhood: cystinuria and cystinosis. Am. J. Med. Sciences 225: 677, 1953.

77. **Silverman, S. H. and Wilkins, L.** Radioiodine uptake in the study of different types of hypothyroidism in childhood. Pediatrics 12: 288, 1953.

78. **Wilkins, L. and Cara, J.** Further studies on the treatment of congenital adrenal hyperplasia with cortisone. V. Effects of cortisone therapy on testicular development. J. Clin. Endocrinol. & Metab. 14:287, 1954.

79. **Wilkins, L., Clayton, G. M. and Berthrong, M.** The development of goiters in cretins without iodine deficiency. Hypothyroidism due to apparent inability of the thyroid gland to synthesize thyroxine. Pediatrics 13: 235, 1954.

80. **Bongiovanni, A.M>.** Detection of corticoid conjugates in human blood. J. Clin. Endocrinol. & Metab. 14: 341, 1954.

80a. **Bongiovanni, A. M., Eberlein, W. R., Grumbach, M. M. Van Wyk, J. J. and Clayton, G.** Conjugates of adrenal corticoids in human plasms. Proc. Soc. Exper. Biol. & Med. 87: 282, 1954.

81. **Wilkins, L., Bongiovanni, A. M., Clayton, G. W., Grumbach, M. M. and Van Wyk, J. J.** The present status of the treatment of virilising adrenal hyperplasia with cortisone. Experience of 3-1/2 years. Mod. Prob. in Ped., S. Karger, Basel, Switz., 1: 329, 1954.

82. **Bongiovanni, A. M., Eberlein, W. R. and Cara, Jose.** Studies on the metabolism of adrenal steroids in the adrenogenital syndrome. J. Clin. Endocrinol. & Metab. 14: 409, 1954.

83. **Bongiovanni, A. M. and Clayton, G. W., Jr.** A simplified method for the routine determination of pregnamedial and pregnanetriol in urine. Bull. Johns Hopkins Hosp. 94: 180, 1954.

84. **Wilkins, L.** Tools and methods of diagnosis and new trends in the treatment of endocrine disorders. (Borden Award Lecture Pediatrics 13: 393, 1954.

85. **Bongiovanni, A. M. and Clayton, G. W., Jr.** A simplified method for the estimation of 11-oxygenated neutral 17-ketosteroids in the urine of individuals with adrenocortical hyperplasia. Proc. Soc. Exper. Biol. & Med. 85: 428, 1954.

86. **Wilkins, L., Bongiovanni, A. M., Clayton, G. W., Grumbach, M. M. and Van Wyk, J. J.** Virilising adrenal hyperplasia: its treatment with cortisone and the nature of the steroid abnormalitites. Ciba Foundation Colloq. Endocrin. 8: 480, 1954.

87. **Wilkins, L., Grumbach, M. M. and Van Wyk, J. J.** Chromosomal sex in ovarian agenesis. J. Clin. Endocrinol. & Metab. 14: 1270, 1954.

1955

88. **Wilkins, L.** The evolution of endocrine diagnosis and treatment. (Addison Lecture) Gay's Hospital Gasette - March, 1955.

88a. **Wilkins, L**. Endocrine Factors in Obesity in Fat Metabolism, ed. by Najjar, Johns Hopkins Press, Baltimore, 1954.

89. **Wilkins, L**. Hormonal influence on skeletal growth. Ann. N. Y. Acad. Sc. 60: 763, 1955.

90. **Grumbach, M. M., Bongiovanni, A. M., Eberlein, W., Van Wy, J. J. and Wilkins, L.** Cushing's Syndrome with bilateral adrenal hyperplasia: a study of the plasma 17-OH-CS and the response to ACTH. Bull. Johns Hopkins Hosp. 96: 116, 1955.

91. **Wilkins, L., Grumbach, M. M., Van Wyk. J. J., Shepard, T. H., II and Papedatos, C.** Hermaphroditism: classification, diagnosis, selection of sex and treatment. Pediatrics 16: 287, 1955.

92. **Grumbach, M. N., Van Wyk, J. J. and Wilkins, L.** Chromosomal sex in gonadal dyagenesis (ovarian agenesis). Relationship to male pseudohermaphroditism and theories of human sex differentiation. J. Clin. Endocrinol. & Metab. 15: 1161, 1955.

93. **Gyorgy, Wilkins, Hampson et al.** Psychologic aspects of sexual orientation of the child with particular reference to the problem of intersexuality. J. Pediat. 47: 771, 1955.

94. **Van Wyk, J. J.** The use of thyroid in pediatric practice. Quart. Rev. Ped. 10: 212, 1955.

95. **Newberry, E. and Van Wyk, J. J.** A technique for quantitative urine collection in the metabolic study of infants and young children. Pediatriecs 16: 667, 1955.

96. **Hampson, Joan G.** Hermaphreditic genital appearane, rearing and eroticism in hyperadrenocorticism. Bull. Johns Hopkins Hosp. 96: 265, 1955.

97. **Hampson, J. L., Hampson, J. G. and Money, J.** The syndrome of gonadal agensis (ovarian agenesis) and male chromosomal pattern in girls and women. Psychologic studies. Bull. Johns Hopkins Hosp: 97: 207, 1955.

98. **Money, J.** Hermaphroditism, gender and precocity in hyperadrenocorticism. Bull. Johns Hopkins Hosp. 96: 253, 1955.

99. **Money, J., Hampson, J. G. and Hampson, J. L.** An examination of some basis sexual concepts: the evidence of human hermaphroditism. Bull. Johns Hopkins Hosp. 97: 301, 1955.

100. **Money, J**. Hermaphroditism: recommendations concerning assignment of sex, change of sex and psychologic management. Bull. Johns Hopkins Hosp. 97: 284, 1955.

100a. **Money, J. and Hampson, J. G**. Idiopathic sexual precocity in the male. Psychosom. Med. 17: 1, 1955.

100b. **Hampson, J. G. and Money, J**. Idiopathic sexual precocity in the female. Psychosom. Med. 17: 16, 1955.

1956

101. **Childs, B., Grumbach, M. M. and Van Wyk, J. J.** Virilising adrenal hyperplasia: a genetic and hormonal study. J. Clin. Invest. 35: 213, 1956.

102. **Wilkins, L.** The influence of the endocrine glands upon growth and development. Chapter IX in Textbook of Endocrinology, 2nd Ed. R. H. Williams, Saunders, 1955.

103. **Van Wyk, J. J., Grumbach, M. M., Shepard, T. H., II and Wilkins, L.** Treatment of hyperthyroidism with thieuracil drugs. Pediatrics 17: 221, 1956.

104. **Grumbach, M. M. and Wilkins, L.** The pathogenesis and treatment of virilizing adrenal hyperplasia. Pediatrics 17: 418, 1956.

105. **Van Wyk, J. J.** Hypothyroidism in childhood. Pediatrics 17: 427, 1956.

106. **Migeon, C. J., Prystowsky, H., Grumbach, M. M. and Byron, M.** Placental passage of 17-OH-CS: comparison of the levels in maternal and fetal plasma and effect of ACTH and hydrocortisone administration. J. Clin. Invest. 35: 488, 1956.

107. **Hinrichs, E. N., Jr.** Dental changes in idiopathic juvenile hypoparathyroidism. Oral Surg., Oral Med. and Oral Path. 9: 1102, 1956.

108. **Money, J., Hampson, J. O. and Hamson, J. L.** Sexual incongruities and psychopathology. The evidence of human hermaphroditism. Bull. John Hopkins Hosp. 98: 43, 156.

109. **Hampson, J. G., Money, J. and Hampson, J. L.** Hermaphroditism, recommendations concerning case management. J. Clin. Endocrinol. & Metab. 16: 547, 1956.

110. **Money, J.** Psychologic studies oin hypothyroidism, recommendations for case management. Arch. Neurol. & Psych. 76: 296, 1956.

1957

111. **Wilkins, L.** The Diagnosis and Treatment of Endocrine Disorders in Childhood and Adolescence. Springfield, Chas. C. Thomas, 1957.

112. **Mosier, H. D.** Comparative histological study of the adrenal cortex of the wild and domesticated Norway rat. Endocrinology 60: 460, 1957.

113. **Wilkins, L.** Presidential Address. May 31, 1957. Endocrinology 61: 206, 1957.

114. **Smith, D. W, Blizzard, R. M. and Wilkins, L.** The mental prognosis in hypothyroidism of infancy and childhood. A review of 128 cases. Pediatrics 19: 1011, 1957.

115. **Migeon, C. J., Bertrand, J. and Well, P. E.** Physiological disposition of 4-C¹⁴-cortisol during late pregnancy. J. Clin. Invest. 36: 1350, 1957.

116. **Migeon, C. J., Keller, A. R., Lawrence, B. and Shepard, T. H.** Dehydroepisndrosterone and androsterone levels in human plasma. Effect of age and sex; day-to-day and diurnal variations. J. Clin. Endocrinol. & Metab. 17: 1051, 1957.

117. **Migeon, C. J., Bertrand, J., Wall, P. E., Stempfel, R. S. and Prystowsky, H.** Metabolism and placental transmission of cortisol during pregnancy near term. Ciba Colloquia on Endocrinology, 1957.

118. **Migeon, C. J. and Stempfel, R. S.** Laboratory diagnosis in pediatric endorinology. Ped. Clin. of N. A., Nov., 1957, page 959, W.B. Saunders Company.

119. **Blizzard, R. M. and Wilkins, L.** Present concepts of steroid therapy in virilizing adrenal hyperplasia. Arch. Int. Med. 100: 729, 1957.

120. **Sandberg, A. A., Eik-Nes, K., Migeon, C. J. and Koepf, G. F.** Plasma 17-hydroxy-corticosteroids in hyperfunction, suppression and deficiency of adrenal cortical function. J. Lab. & Clin. Med. 50: 286, 1957.

121. **Bliss, E. L. and Migeon, C. J.** Endocrinology of anorexia nervosa. J. Clin. Endocrinol. & Metab. 17: 766, 1957.

1958

122. **Mosier, H. D. and Richter, C.P.** Response of the glomerulosa layer of the adrenal gland of wild and domesticated Norway rats to low and high salt diets. Endocrinology 62: 268, 1958.

123. **Mosier, H. D., Blizzard, R. M. and Wilkins, L.** Congenital defects in the biosynthesis of thyroid hormone. Report of two cases. Pediatrics 21: 248, 1958.

124. **Stempfel, R. S. and Migeon, C.J.** Precocious and delayed sexual development. Clin. Ob. and Gyn. 1: 271, 1958.

125. **Howard, J. E. and Migeon, C. J.** Cushing's Syndrome produced by normal replacement doses of cortisone in a patient with defective mechanism of steroid degradation. Am. J. Med. Sci. 235: 387, 1958.

126. **Wilkins, L., Jones, H. W., Jr., Holman, G. H. and Stempfel, R. S., Jr.** Masculinization of the female fetus associated with administration of progestins during gestation: Non-adrenal female pseudohermaphroditism. J. Clin. Endocrinol. & Metab. 18: 559, 1958.

127. **Nichols, J., Lescure, O. L. and Migeon, C. J.** The levels of 17-hydroxy-corticosteroids and 17-ketosterodis in maternal and cord plasma in term anencephaly. J. Clin. Endocrinol. & Metab. 18: 444, 1958.

128. **Martin, M. M. and Wilkins, L.** Pituitary dwarfism: Diagnosis and treatment. J. Clin. Endocrinol. & Metab. 18: 679, 1958.

129. **Wilkins, L.** Dysgenesis gonadale et hermaphrodisme. Leurs relations avec les theories de la differenciation sexuelle. Marseille Medical 95: 1, 1958.

130. **Wilkins, L**. Syndrome Adreno-genital. Les Ann. d'Endocrin. 19: 841, 1958.

131. **Stempfel, R. S., Jr., Sidbury, J. B., Jr. and Migeon, C. J.** The effects of large doses of salicylate on the metabolism of cortisol in human subjects. (Abstract) Am. J. Dis. Child. 96: 543 , 1958.

132. **Holman, G. H. and Migeon, C. J.** A functional ensymatic deficiency of cortisol metabolism in young infants. (Abstract) Am. J. Dis. Child. 96: 524, 1958.

1959

133. **Migeon, C. J., Wall, P. E. and Bertrand, J.** Some aspects of the metabolism of 16-C¹⁴-esterone in normal individuals. J. Clin. Invest. 38: 619, 1959.

134. **Wall, P. E. and Migeon, C. J.** In vitro studies with 16-C^14^-estrone: distribution between plasma and red blood cells of man. J. Clin. Invest. 38: 611, 1959.

135. **Gardner, L. I. and Migeon, C. J.** Unusual plasma 17-ketosteroid pattern in a boy with congenital adrenal hyperplasia and periodic fever. J. Clin. Endocrinol. & Metab. 19: 266, 1959.

136. **Childs, B., Sidbury, J. B., Jr. and Migeon, C. J.** Glucuronic acid conjugation by patients with familial non-hemolytic jaundice and their relatives. Pediatrics 23: 903, 1959.

137. **Haddad, H. M. and Wilkins, L.** Congenital anomalies associated with gonadal aplasia. Pediatrics 23: 885, 1959.

138. **Blizzard, R. M., Liddle, G. W., Migeon, C. M. and Wilkins, L.** Aldosterone excretion in patients with virilizing adrenal hyperplasia maintained on a normal and low salt diet. J. Clin. Invest. (In press).

139. **Howard, E. and Migeon, C. J.** Sex hormone secretion by the adrenal cortex. In The Adrenocortical Hormones: their chemistry, physiology and pharmacology. Springer Verlag, Editor. (In press).

140. **Migeon, C.J.** Androgens in human plasma. In Symposium on Hormones in Human Plasma. Antoniades, H., Editor (In press).

141. **Migeon, C.J., Lawrence, B., Bertrand, J. and Holman, G. H.** In vivo distribution of some 17-hydroxycorticosteroids between plasma and red blood cells of man. J. Clin. Endocrinol. & Metab. (Submitted).

142. **Migeon, C.J., Lesource, O. and Antoniades, H.** Further in vitro studies with 16-C¹⁴-estrone: distribution between plasma protein fractions and red blood cells of man. J. Clin. Invest. (Submitted).

143. **Migeon, C.J.** Cortisol production and metabolism in the neonate. A.M.A.J. Pediatrics (In press).

144. **Wilkins, L.** Masculinization of the female fetus due to the use of certain synthetic oral progestins during pregnancy. Arch. d'Anat. Mier. et Morph. Exp. (In press).

145. **Haddad, Heskel M. and Sidbury, J.B., Jr.** Defect of the Iodinating system in congenital goitrous cretinism: report of a case with biochemical studies. J. Clin. Endocrinol. & Metab. 19: 1446, 1959.

145a. **Albert, A., Burns, E., Hampson, J.** Determination of sex and what to do about it. J. Urol. 81: 13, 1959.

1960

146. **Wilkins, L.** Abnormalities of Sex Differentiation: Classification, Diagnosis, Selection of Gender of Rearing and Treatment. Pediatrics 26: 846, November, 1960.

147. **Wilkins, L**. The thyroid gland. Scientific American 202: 119, 1960.

148. **Wilkins, L**. Masculinization of Female Fetus due to Use of Orally given Progestins. J.A.M.A. 172: 1028, 1960 (March 5).

149. **Wilkins, L**. Diagnosis, Selection of Sex of Rearing and Treatment of Various Types of Abnormal Sex Differentiation. In Clinical Endocirnology, I (N.Y., Grune & Stratton). 437-454, 1960.

150. **Wilkins, L**. Hypothyroidism in Children. In Clinical Endocrinology, I (N.Y., Grune & Stratton). 112-122, 1960.

151. **Green, O.C., Migeon, C.J., and Wilkins, L.** Urinary steroids in the hypertensive form of congenital adrenal hyperplasia. J. Clin. Endocrinol. 20: 929, July, 1960.

152. **Wilkins, L.** The Influence of the Endocrine Glands upon Growth and Development, in Textbook of Endocrinology, 3rd edn., R. H. Williams, Saunders, 1960.

153. **Migeon, C.J., Nicolopoulos, D., and Cornblath, M.** Concentrations of 17-hydroxycorticosterodis in the blood of diabetic mothers and in blood from the umbilical cords of their offspring at the time of delivery. Pediatrics 25: 605, 1960.

154. **Stempfel, R.S., Jr., Sidbury, J. B., Jr., and Migeon, C.J.** *B*-Glucuronidase hydrolysis of urinary corticosteroid conjugates: Effect of salicylate glucuronidase as a competing enzyme of enzyme inactivation. JCE&M 20: 814, 1960.

155. **Haddad, Heskel M., and Jones, H.W.** Clitoral Enlargement stimulating pseudohermaphroditism. AMA Journal of Diseases of Children 99: 282, 1960.

156. **Cleveland, W., Green, O.C., Migeon, C.** A Case of Proved Adrenocorticotropin Deficiency. Journal of Ped. 57: 376, 1960.

156a. **Migeon, C.J.** Androgens in Human Plasma. Hormones in Human Plasma edited by Antoniades, H. Little Brown and Company, Boston, Mass., 1960.

156b. **Issacs, James P., Blalock A., and Migeon, C. J.** Catecholamine and 17-hydroxycorticosteroid output in dogs with transplanted adrenal glands. Bull. JHH 107: 105, August, 1960.

157. **Park, E.A. and Bongiovanni, A.** Biographies of Wilkins. J. Pediatrics, September, 1960.

158. **Jones, H., Wilkins, L.** The Genital Anomaly with Prenatal Exposure to Progestogens. Fertility and Sterility 11: 148, March-April, 1960.

159. **Blizzard, R. M.** Inherited Defects of Thyroid Hormone Synthesis and Metabolsim. Metabolism 9: 232, March, 1960.

160. **Green, R. Money, J.** Incongruous Gender Role: Nongenital Manifestations in Prepurbertal Boys. The J. of Nervous and Mental Diseases, 130: 160, August, 1960.

161. **Money, J.** Components of Eroticism in Man: Cognitional Rehearsals. Recent Advances in Biological Psychiatry. 210, 1960.

1961

162. **Wilkins, L.** Diagnosis and Treatment of Congenital Virlizing Adrenal Hyperplasia. Postgraduate of Medicine 29: 31, 1961.

163. **Shulman, L.E., Calkins, E., Cluff, L., and Wilkins, L.** Adrenocortical Steroid Therapy (A Panel Discussion) Md. State Med.J., May, 1961.

164. **Green, O.C., Cleveland, W.W., and Wilkins, L.** Triamcinolone therapy in the adrenaogenital syndrome. Pediatrics 27: 292, February, 1961.

165. **David, R. R., Alexander, D.S. and Wilkins, L.** Placental Transfer of an organic radiopaque medium resulting in a prolonged elevation of the protein bound iodine. J. of P. August, 1961, 59: 223.

166. **Money, J.** Hermaphroditism. The Encyclopedia of Sexual Behavior. 472, 1961.

167. **Green, R., Money, J.** Effeminacy in Prepubertal Boys. Summary of 11 Cases and Recommendations for Case Management. 27: 286, February, 1961.

168. **Money, J.** Components of eroticism in Man. I. The hormones in relation to sexual morphology and sexual desire. J. Nerv. and Mental Dis. 132: 239, 1961.

169. **Migeon, C. J., Bertrand, J., Gemzell, C.A.** The Transplacental Passage of Varioous Steroid Hormones in Mid Pregnancy. Present Progress in Hormone Research. 17: 207, 1961.

170. **Migeon, C.J.** The Endocrine Function of the Newborn. CIBA Found. Symposium on Somatic Stability in the Newly Born. 1961. P. 215-237.

171. **Migeon, C. J., Bertrand, J. and Gemzell, C. A.** The Transplacental Passage of Various Steroid Hormones in Med Pregnancy. Recent Progress in Hormone Research. 17: 207, 1961. Also in the Human Adrenal Cortex. Currie Symington and Grant, Editors, Edinburgh, London, 1962.

172. **Cleveland, W. W., Green, O.C., and Wilkins, L.** Deaths in Congenital Adrenal Hyperplasia. Pediatrics. 29: 3, January, 1962.

173. **Aceto, T., Blizzard, R., and Migeon, C.J.** Adrenocortical Insufficiency in Infants and Children. The Ped. Clinics of N. Amer. 9: 1, Feb., 1962.

174. **Wilkins, L.** The Effects of Thyroid Deficiency Upon the Development of the Brain. Vol. XXXIX. P. 150-155. Research in Nervous and Mental Disease, 1962.

175. **Cleveland, W.W., Nikesic, M., and Migeon, C.J.** Response to an 11-Hydroxylase Inhibitor (SU-4885) in Patients with Adrenal Hyperplasia and Their Parents. J. Clin. Endocrinol. Metab 22: 281, 1962.

176. **Howard, E. and Migeon, C.J.** Sex Hormone Secretion By the Adrenal Cortex. Handbuch Der Experimentallen Pharmakologie Vol. 14: 560-624. Springer-Verlag, Heidelberg, 1962.

177. **Wilkins, L.** Adrenal Disorders. I. Cushing's Syndrome and Its Puzzles. Arch. Dis. Childhood 37: 1, 1962. II. Virilizing Adrenal Hyperplasia. Arch. Dis. Childhood 3: 231, 1962.

178. **Bergada, C., Cleveland, W. W., Jones, H. W., and Wilkins, L.** Gonaldal Histology in Patients with Male Pseudohermaphroditism and Atypical Gonadal Dysgenesis: Relation to Theories of Sex Differentiation. Acta Endocrinologica. 40: 493-520, 1962.

179.**Bergada, C., Cleveland, W. W., Jones, H. W., and Wilkins, L.** Variants of Embryonic Testicular Dysgenesis: Bilateral Anorchia and the Syndrome of Rudimentary Testes. Acta Endocrinologica. 40: 521-536, 1962.

180. **Hung, W., Wilkins, L. and Blizzard, R.M.** Medical Therapy of Thyrotoxicosis in Children. Pedatrics 30: 1, July 1962.

181. **Chandler, R. W., Kyle, M. A., Hung, W., and Blizzard, R. M.** Experimentally Induced Autoimmunization Disease of the Thyroid. I. The Failure of Transplacental Transfer of Anti-Thyroid Antibodies to Produce Cretinism. 40:6, Pediatrics, June 1962.

182. **Oh, W., Baens, G. S., Migeon, C. J., Wybregt, S. H., and Cornblath, M.** Studies of Carbohydrate Metabolism in the Newborn Infant. V. The Effects of Cortisol on the Hyperglycemic Response to Glucagon. Pediatrics 30: 763, 1962.

183. **Rappaport, R., and Migeon, C. J.** Physiological Disposition of 4-C^14^-Tetrahydrocortisol in Man. J. Clin. Endocrinol. Metab. 22: 1065, 1962.

184. **Migeon, C. J., Lescure, O. L., Zinkham, W. H., and Sidbury, J. B.** In Vitro Interconversion of 16-C^14^-Estrone and 16-C^14^-Estradiol-17 β by Erythrocytes from Normal Subjects and From Subjects with **a** Deficiency of Red Cell Glucose-6-Phospate Dehydrogenase Activity. J. Clin. Invest. 41: 2025, 1962.

Also presented at the First International Congress of Endocrinology, Copenhagen, 1960.

185. **Wilkins, L.** Modern Materia Medica (Presidential Address). American Pediatric Society. Am. J. Dis. Child 104: 449, 1962.

1963

186. **Kenny, F. M., Malvaux, P., and Migeon, C. J.** Cortisol Production Rate in Newborns, Infants, and Children. Pediatrics 31: 360, 1963.

Also presented at the 32nd meeting of the Society for Pediatric Research, Atlantic City, May 8-10, 1962. AMA J. Dis. Child 104: 529, 1962.

187. **Migeon, C. J., Green, O.C., and Eckert, J. P.** Study of Adrenocortical Function in Obesity. Metabolism 12: 718, 1963.

Also presented at the 43rd meeting of the Endocrine Society, New York, June 1961.

188. **Camacho, A. M. and Migeon, C. J.** Isolation, Identification, and Quantification of Testosterone in the Urine of Normal Adults and in Patients with Endocrine Disorders. J. Clin. Endocrinol. l Metab. 23: 301, 1963.

Also presented at the 44th meeting of the Endocrine Society, Chicago, June 1962.

189. **Hung, W., Migeon, C. J., and Parrott, R.M.** A Possible Autoimmune Basis for Addison's Disease in Three Siblings, One with Idiopathic Hypoparathyroidism, Pernicious Anemia, and Superficial Moniliasis. New Eng., J. Med 269: 658, 1963.

190. **Hung, W., Blizzard, R. M., Migeon, C. J., Nyhan, W. and Comacho, A.M.** Precocious Puberty in a Boy with a Hepatoma and Circulating Gonadotropin. J. Pediatrics 63: 895, 1963.

1964

191. **Camacho, A. M. and Migeon, C. J.** Studies of the Origin of Testosterone in the Urine of Normal Adult Subjects and Patients with Various Endocrine Disorders. J. Clin. Invest. 43: 1083, 1964.

192. **Kowarski, A., Finkelstein, J. W., Loras, B., and Migeon, C. J.** The In Vivo Stability of the Tritium Label in 1, 2-H³-Aldosterone Secretion Rate by the Double Isotope Dilution Technique. Steroids 2: 95, 1964.

1965

193. **Finkelstein, J. W., Kowarski, A., Spaulding, J. S., and Migeon, C. J.** Effect of Various Preparations of Human Growth Hormone on Aldosterone Secretion Rate of Hypopituitary Dwarfs. Am. J. Med. 38: 517, 1965.

194. **David, R. R., Bergada, C., and Migeon, C. J.** Isolation, Identification and Measurement of 3α, 17α-Dihydroxypregnane-11, 20-Dione in Congenital Adrenal Hyperplasia. J. Clin. Endocrinol. Metab. 25: 322, 1965.

195. **Bowen, P., Lee, C.S.N., Migeon, C. J., Kaplan, N. M., Whalley, P.J., McKusick, V. A., and Reifenstein, E. C.** Hereditary Male Pseudohermaphroditism with Hypogonadism, Hypospadias and Gynecomastia (Reifenstein's Syndrome). Annals of Internal Medicine 62: 252, 1965.

196. **Aarskog, D., Blizzard, R. M. and Migeon, C. J.** The Response to Methopyrapone (SU-4885) and Pyrogen Tests in Idiopathic Hypopituitary Dwarfism. J. Clin. Endocrinol. Metab. 25: 439, 1965.

197. **David, R. R., Bergada, C., and Migeon, C. J.** Effect of Age on Urinary Steroid Excretion in Congenital Adrenal Hyperplasia. Bull. Johns Hopkins Hosp. 117: 16, 1965.

198. **Kowarski, A., Finkelstein, J. W., Spaulding, J. S., Holman, G. H., and Migeon, C. J.** Aldosterone Secretion Rate in Congenital Adrenal Hyperplasia. J. Clin. Invest. 44: 1505, 1965.

199. **Migeon, C. J. and Baulieu, E.** Hyperplasie Congenitale Des Surrenales-Etde Biologique. Les Trouble Congenitaux de l'hormogenese. Doin and Masson Ed. Paris, 1965. Presented at VIII Reunion des Endocrinologistes deLangue Francaise, Paris, June 1965.

200. **Kenny, F. M., Preeyasombat, C., and Migeon, C. J.** Cortisol Production Rate. II. Normal Infants, Children, and Adults. Pediatrics 37: 34, 1966.

201. **Rivarola, M. A. and Migeon, C. J.** A Method for the Determination of Cortisol Secretion Rate in Patients Receiving Antibiotics. Bull. Johns Hopkins Hosp. 17: 286, 1965.

202. **Snipes, C. A., Becker, W. G., and Migeon, C. J.** The Effect of Age on the In Vitro Metabolism of Androgens by Guinea Pig Testies. Steroids 6: 771, 1965.

1966

203. **Kowarski, A., Bernant, M., Grossman, M.S. and Migeon, C. J.** Antidiuretic Property of Aldactone (spironolactone) in Diabetes Insipidus. Studies on the Mechanism of Antidiuresis. Bull. Johns Hopkins Hosp. 119: 413, 1966.

204. **Kenny, F. M., Preeyasombat, C., Spaulding, J. S. and Migeon, C. J.** Cortisol Production Rate. IV. Infants Born of Steroid-Treated Mothers and of Diabetic Mothers. Infants with Trisomy Syndrome and with Anencephaly. Pediatrics 37: 960, 1966.

205. **Becker, W. G., Snipes, C. A. and Migeon, C. J.** Progesterone-4-C¹⁴. Metabolism to Androgens by Testes of Normal and Isoimmune Aspermatogenic Guinea Pigs. Endocrinology 78: 737, 1966.

206. **Rivarola, M. A. and Migeon, C. J.** Determination of Testosterone and Androst-4-Ene-3, 17-Dione Concentration in the Human Plasma. Steroid 7: 103, 1966.
